# Lower hypothalamus subunit volumes link with impaired long-term body weight gain after preterm birth

**DOI:** 10.3389/fendo.2022.1057566

**Published:** 2022-12-15

**Authors:** Tobias Ruzok, Benita Schmitz-Koep, Aurore Menegaux, Robert Eves, Marcel Daamen, Henning Boecker, Esther Rieger-Fackeldey, Josef Priller, Claus Zimmer, Peter Bartmann, Dieter Wolke, Christian Sorg, Dennis M. Hedderich

**Affiliations:** ^1^ Department of Neuroradiology, Klinikum rechts der Isar, School of Medicine, Technical University of Munich, Munich, Germany; ^2^ TUM-NIC, Technical University of Munich Neuroimaging Center, Munich, Germany; ^3^ Department of Psychology, University of Warwick, Coventry, United Kingdom; ^4^ Department of Psychology, Bielefeld University, Bielefeld, Germany; ^5^ Clinical Functional Imaging Group, Department of Diagnostic and Interventional Radiology, University Hospital Bonn, Bonn, Germany; ^6^ Department of Neonatology, University Hospital Bonn, Bonn, Germany; ^7^ Department of Neonatology, Klinikum rechts der Isar, School of Medicine, Technical University of Munich, Munich, Germany; ^8^ Department of Psychiatry and Psychotherapy, Klinikum rechts der Isar, School of Medicine, Technical University of Munich, Munich, Germany; ^9^ Neuropsychiatry, Charité - Universitätsmedizin Berlin and German Center for Neurodegenerative Diseases (DZNE), Berlin, Germany; ^10^ UK Dementia Research Institute, University of Edinburgh (UK DRI), Edinburgh, United Kingdom; ^11^ Warwick Medical School, University of Warwick, Coventry, United Kingdom

**Keywords:** body weight gain, hypothalamus subunits, preterm birth, small for gestational age, catch-up growth, magnetic resonance imaging

## Abstract

**Introduction:**

Preterm birth is associated with an increased risk for impaired body weight gain. While it is known that in prematurity several somatic and environmental factors (e.g., endocrine factors, nutrition) modulate short- and long-term body weight gain, the contribution of potentially impaired body weight control in the brain remains elusive. We hypothesized that the structure of hypothalamic nuclei involved in body weight control is altered after preterm birth, with these alterations being associated with aberrant body weight development into adulthood.

**Materials and methods:**

We assessed 101 very preterm (i.e., <32 weeks of gestational age) and/or very low birth weight (i.e., <1500g; VP/VLBW) and 110 full-term born (FT) adults of the population-based Bavarian Longitudinal Study with T1-weighted MRI, deep learning-based hypothalamus subunit segmentation, and multiple body weight assessments from birth into adulthood.

**Results:**

Volumes of the whole hypothalamus and hypothalamus subunits relevant for body weight control were reduced in VP/VLBW adults and associated with birth variables (i.e., gestational age and intensity of neonatal treatment), body weight (i.e., weight at birth and adulthood), and body weight trajectories (i.e., trajectory slopes and cluster/types such as long-term catch-up growth). Particularly, VP/VLBW subgroups, whose individuals showed catch-up growth and/or were small for gestational age, were mostly associated with volumes of distinct hypothalamus subunits such as lateral or infundibular/ventromedial hypothalamus.

**Conclusion:**

Results demonstrate lower volumes of body weight control-related hypothalamus subunits after preterm birth that link with long-term body weight gain. Data suggest postnatal development of body weight -related hypothalamic nuclei in VP/VLBW individuals that corresponds with distinct body weight trajectories into adulthood.

## 1 Introduction

Preterm birth is defined as birth before 37 weeks of gestational age (GA) and is frequent with a worldwide prevalence of about 11% (1). Preterm birth is associated with increased risks for somatic, behavioral, and neuro-cognitive impairments, such as metabolic or cardio-vascular disorders (2, 3) or lower IQ (4, 5). Concerning metabolic impairments, preterm birth elevates the risk for impaired body weight gain, with increasing risks for those born very preterm (i.e., <32 GA) and/or with very low birth weight (i.e., <1500g; VP/VLBW); impaired postnatal to long-term body weight gain is broadly defined as the failure to achieve the body weight gain-potential expected for an individual at a certain age (6–8). While recent studies suggest that progress in modern neonatal management contributes to overcome postnatal differences in body height of VP/VLBW compared to full-term (FT; i.e., ≥37 GA and ≥1500g) born infants until childhood, body weight differences are not yet compensated (9). Particularly, VP/VLBW infants born below the 10th percentile of weight for their gestational age (i.e., born small for gestational age, SGA) have larger hazards for impaired postnatal body weight gain and/or impaired long-term catch-up growth, which, in turn, is associated with increased risks for long-term morbidity (8, 10, 11). Indeed, impaired body weight gain of, for example, preterm infants, links with increased risks for adverse outcomes - from neurodevelopmental aberrations to aberrant glucose tolerance and diabetes mellitus-type-II (12–14). Moreover, impaired body weight gain represents a complex and multifactorial condition modified by maternal, genetic, fetal, environmental, nutritional, stress-related, and endocrine factors (10, 11, 15). At the same time, also rapid gain of body weight during infancy (early catch-up growth), especially for those being SGA, is seen as an independent risk factor for e.g., obesity or hypertension later in life (11, 16–18). While our knowledge about environmental to somatic factors is remarkable, we still do not know, however, whether altered brain mechanisms of body weight control are also associated with aberrant body weight development after preterm birth.

The hypothalamus is critically involved in growth control, including control of body weight (19–21). It is a highly conserved brain structure across vertebrates, surrounding the infundibular recess of the forebrain’s third ventricle and consisting of at least 13 interconnected nuclei (22). Although neurogenesis of the hypothalamus starts already during gestational week 9 in humans (23), its development, ranging from neuronal migration, axon extension, dendritic arborization and synaptogenesis to myelination and epigenetic modifications, is not terminated prenatally, but includes subsequent postnatal development (24). It is known that the hypothalamus controls a variety of basic physiological-behavioral processes, from circadian rhythms to drinking, feeding, sexual, and threat behavior (21). In particular, several hypothalamic nuclei, including the paraventricular (PVN), infundibular (INF), dorso-/ventromedial nucleus (DM/VM), and the lateral hypothalamus (LH), are specifically involved in the control of appetite, food intake, and growth, including body weight (20, 25, 26). While most evidence about hypothalamic functions stems from animal studies (e.g., (27)), neuroimaging studies in humans have linked altered hypothalamic structure with impaired body weight control e.g., in obesity and anorexia nervosa (28, 29).

**Figure 1:**
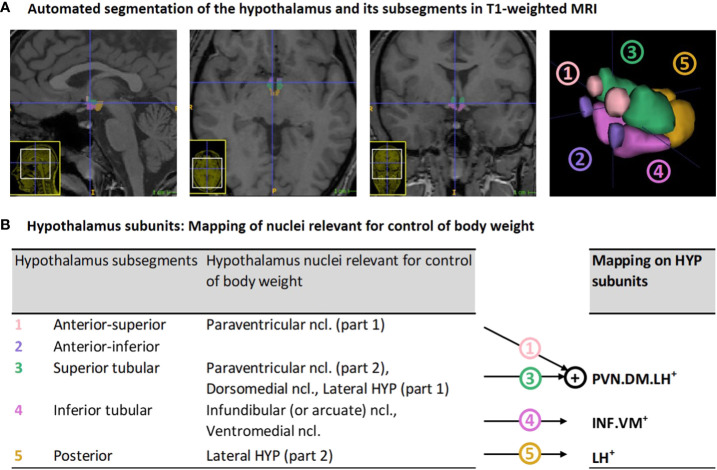
Hypothalamus segmentation and mapping of nuclei relevant for control of body weight on segmentation-based subunits: **(A)** Representative segmentation of the hypothalamus of a VP/VLBW adult *via* deep convolutional neural network algorithm in T1-weighted MRI. From left to right: sagittal, axial, coronal view, and 3D rendering of the whole hypothalamus and its subsegments. **(B)** Mapping of segmentation-based hypothalamic subsegments that contain nuclei relevant for control of body weight (segmentation algorithm based on Billot et al. (33)) onto three *newly defined subunits of body weight control, namely PVN.DM.LH^+^, INF.VM^+^ and LH^+^*. HYP, hypothalamus; MRI, magnetic resonance imaging; VP/VLBW, very preterm and/or very low birth weight.

*In-vivo* imaging of the hypothalamus at a nuclear level in humans is challenging due to hypothalamus small size of about 1cm³ and surrounding gray matter (30, 31). It therefore requires high spatial resolution imaging and, particularly, optimal delineation methodology. While previous approaches on delineation relied on manual, semi-automated or automated Bayesian or multi-atlas segmentation techniques (see overview in (32)), recent improvements in deep learning-based segmentation (i.e., automated deep convolutional neural networks) (33) enable highly reliable hypothalamus delineation, sensitive to inter-individual differences and, most importantly, sensitive to the identification of hypothalamus subsegments that can be mapped on nuclei that are relevant for body weight control (**Figure 1**).

We assessed the role of the hypothalamus in body weight control in VP/VLBW individuals by addressing the following hypotheses: After preterm birth, (i) the structure of whole hypothalamus and hypothalamic nuclei involved in body weight control is altered, and (ii) these alterations are associated (a) with aberrant body weight development into adulthood, particularly in individuals (b) with long-term catch-up growth and (c) born SGA. To test these hypotheses, we assessed 101 VP/VLBW and 110 FT adults with T1-weighted MRI, deep learning-based hypothalamus segmentation, hypothalamic volume as proxy for hypothalamic structure, and multiple body weight assessments, including both slope and cluster/type analysis of long-term body weight trajectories, from birth to adulthood.

## 2 Materials and methods

### 2.1 Participants

Data for this study were derived from the Bavarian Longitudinal Study, BLS, a prospectively geographically defined whole population study of neonatal at-risk VP/VLBW and FT individuals, described in detail in (34–36) and in the Supplement. Briefly, neonatal at-risk children and healthy controls from southern Bavaria were included between January 1985 and March 1986. 682 infants born VP/VLBW and 350 of 916 randomly selected FT controls from the same obstetric hospitals were enrolled and matched for sex and socioeconomic status. Of the initial 682 VP/VLBW infants, 411 were eligible for the 26-year follow-up assessment, and 260 (63.3%) participated in psychological assessments (37). Of the initial 350 FT born infants, 308 were eligible for the 26-year follow-up assessment, and 229 (74.4%) participated in psychological assessments. Of the sample assessed in adulthood, 101 VP/VLBW and 111 FT individuals underwent MRI at 26 years of age. MR-related exclusion criteria included (self-reported) claustrophobia, inability to lie still for > 30min, unstable medical conditions (e.g., severe asthma), epilepsy, tinnitus, pregnancy, non-removable MRI-incompatible metal implants, and a history of severe central nervous system (CNS) trauma or disease that would impair further analysis of the data. The most frequent reason not to perform the MRI exam, however, was lack of motivation. **Supplement Figure S1** shows participant flowchart. For comparability of MRI-assessed VP/VLBW and FT groups with respect to brain abnormalities such as ventricle enlargement or neonatal intraventricular hemorrhage, see Supplement Methods and **Table S1**. MRI took place at two sites, namely Department of Neuroradiology, Klinikum rechts der Isar, Technical University of Munich (n=146), and Department of Radiology, University Hospital of Bonn (n=66).

### 2.2 Birth-related variables, neurocognitive assessment, and body weight development

We performed canonical measurements of birth-related variables (gestational age (GA), medical treatment at birth, family socioeconomic status (SES)), full-scale intelligence quotient (FS-IQ; for neurocognitive assessment), and body weight development, described in detail previously (36) and in the Supplement. Briefly, GA was estimated from maternal reports on the first day of the last menstrual period and from serial ultrasounds during pregnancy. To estimate medical impairments at birth, Intensity of Neonatal Treatment Index (INTI) was calculated *via* daily assessments of care level, respiratory support, feeding dependency, and neurological status (including mobility, muscle tone, and neurological excitability; **Supplementary Table S2** for variable description). Family socioeconomic status (SES) at birth was collected through structured parental interviews within 10 days of childbirth. It was computed as a weighted composite score based on the profession of the self-identified head of each family together with the highest educational qualification held by either parent (38). Participants’ neurocognitive functioning at 26 years of age was assessed using the short version of the German Wechsler Adults Intelligence Scale, Third edition (WAIS-III) (39). The assessment took place prior to and independent of the MRI scan and was carried out by trained psychologists who were blinded to group membership. Consecutively, full-scale intelligence quotient (FS-IQ) performance was computed.

Longitudinal body weight measurements were undertaken at birth, at five and 20 months corrected for prematurity, at 56 months, 6, 8 and 26 years of chronological age, using predefined protocols (40). For body weight development analysis, we transformed body weight measurements from [g]/[kg] into z-scores relative to the exact age of each participant, typically used for the description of longitudinal changes of growth. Applied references for calculation of z-scores are described in the Supplement methods. “Small for gestational age” (SGA) versus “appropriate/large for gestational age” (AGA/LGA) was determined by birth weight (BW) z-scores being below (equivalent to z-scores <-1.282) versus above the 10th percentile, respectively (41), with a relevant number of SGA-born individuals only in the VP/VLBW (n=30) but not in the FT group (n=8). Within the SGA cohort, successful/failed endpoint catch-up growth was defined as an adult body weight z-score at age 26 above/below the 10th percentile i.e., we focused on *long-term* body weight gain (6). For body weight trajectory analysis, we performed both trajectory slope and trajectory type analysis using Python version 3.7.10 and especially the “scikit-learn” package, a machine learning focused Python library. For trajectory slope analysis, linear regression of longitudinal body weight z-scores (non-interpolated) was performed to receive regressed body weight trajectories; change of body weight z-scores from birth until adulthood of regressed body weight trajectories defined long-term delta slopes. For trajectory type analysis, we clustered VP/VLBW body weight trajectories *via* k-means algorithm in Python (sklearn.cluster.KMeans). The number of times the k-means algorithm was run with different centroid seeds as default initialization was n=100.

We additionally undertook an exploratory analysis to identify the relationship between adult hypothalamic volumes and early catch-up (or catch-down) growth in body weight. Following the literature, early catch-up growth was defined as an increase of z-score of at least 0.67 during the first two years of life, with early catch-down representing a decrease of 0.67, respectively (16, 42). We used the first three measurements of body weight z-scores within our dataset (birth, five and 20 months) to perform linear regression equivalent to the long-term slope analysis; change of body weight z-scores from birth until the age of 2 years of those regressed body weight trajectories defined “short-term delta slope” (defining: “early catch-up” if “short-term delta slope” > 0.67; “early catch-down” if “short-term delta slope” < -0.67).

### 2.3 MRI data acquisition and hypothalamus segmentation

MRI data acquisition (for details see (36)) was performed on Philips Achieva 3T systems or Philips Ingenia 3T systems using an 8-channel SENSE head coil. Subject distribution among scanners was: Bonn Achieva 3T: 5 VP/VLBW, 11 FT; Bonn Ingenia 3T: 33 VP/VLBW, 17 FT; Munich Achieva 3T: 60 VP/VLBW, 65 FT; Munich Ingenia 3T: 3 VP/VLBW, 17 FT. Across all scanners sequence parameters were kept identical, namely high-resolution T1-weighted 3D magnetization prepared rapid acquisition gradient echo sequence with TI=1.3ms, TR=7.7ms, TE=3.9ms, flip angle=15°, 180 sagittal slices, FOV=256×256×180mm, reconstruction matrix=256×256, and reconstructed isotropic voxel size=1mm³. To account for possible confounds by scanner differences, MRI data analyses included scanner dummy variables as covariates of no interest.

T1-weighted MRI scans in Nifti-format were processed by using FreeSurfer version 7.2 (http://surfer.nmr.mgh.harvard.edu/), which includes a deep convolutional neural network tool of Billot et al. (33) that enables for automated segmentation of the hypothalamus, including subsegment parcellation (**Figure 1A**). To ensure the reliability of the applied hypothalamic segmentation, we compared measured hypothalamic volumes with those of previous hypothalamus parcellation studies (see **Supplementary Figure S2** and **Table S3**). We found similar hypothalamic volumes for our FT cohort, supporting the reliability of the hypothalamic segmentation.

Estimation of total intracranial volume (TIV; sum of segmented gray and white matter brain volumes and cerebrospinal fluid partitions) was performed with the CAT12 toolbox, version r1364 (http://www.neuro.uni-jena.de/cat/) (43) within SPM12 (https://www.fil.ion.ucl.ac.uk/spm/software/spm12/).

To focus on hypothalamic nuclei involved in body weight control, we mapped subsegments from the segmentation algorithm that contained nuclei relevant for body weight control onto three so-called subunits of body weight control, namely PVN.DM.LH^+^, INF.VM^+^ and LH^+^ (see **Figure 1B**). The PVN.DM.LH^+^ subunit is the aggregate of two subsegments of the segmentation of Billot et al. (33), namely the anterior-superior and superior tubular subsegment, and these two subsegments cover the preoptic area, the paraventricular nucleus (PVN), the dorsomedial nucleus (DM), and parts of the lateral hypothalamus (LH); the latter three nuclei are involved in body weight control, therefore defining the name of the subunit. INF.VM^+^ is identical to the inferior tubular subsegment, which comprises - amongst other nuclei - the body weight control-related infundibular (INF) and ventromedial (VM) nuclei. LH^+^ matches the posterior subsegment in Billot et al. (33), including mammillary bodies, and parts of the tuberomammillary nucleus, and, critically, of the body weight control-related lateral hypothalamus (LH). The final anterior-inferior subsegment (suprachiasmatic nucleus and parts of the supraoptic nucleus) was excluded from further analysis because it does not contain any body weight control-related nuclei. All subunits stated in the analysis already consider volumes of bilateral hypothalamus.

### 2.4 Statistical analysis

Statistical analyses were performed using SPSS version 27 (IBM SPSS Statistics). Regarding demographical characteristics, group differences between VP/VLBW and FT cohorts were assessed using chi-square tests (sex, SES) and two-sample t-tests (age, GA, FS-IQ). To test whether both hypothalamic volumes and body weights are altered in prematurity, general linear models were used (dependent variable: hypothalamic volumes or body weights; fixed factor: status of prematurity; covariates: sex and additionally only for hypothalamic volume changes: scanner, TIV), thus controlling for differences in head size, gender and subject-to-scanner distribution. We did, however, not account explicitly for additional perinatal and gestational factors also affecting outcomes of preterm birth, including those on the brain (comp. chapter “Strengths and limitations”), because we were primarily interested in the general relationship between preterm birth, body weight development, and hypothalamus. Partial correlation analysis, restricted to the VP/VLBW group and corrected for sex, scanner and TIV, was used to investigate the associations between hypothalamic volumes and variables of preterm birth or body weight trajectories, respectively. To assess potential mediation effects of hypothalamic volumes regarding the association of variables of prematurity (i.e., INTI and GA, respectively) with adult body weight, a mediation analysis restricted to the VP/VLBW cohort was performed using the PROCESS toolbox (version 3.5) of SPSS.

As adult body weight at age 26 was missing for four individuals of the VP/VLBW cohort, they were excluded from all analyses related to body weight. For trajectory type analysis (clustering of body weight trajectories) missing longitudinal body weight data points were approximated *via* linear interpolation (compare Supplement methods for more detailed information).

Statistical significance was set at p <0.05; all tests were two-sided. Tests were corrected for multiple comparisons for false discovery rate (FDR) according to the Benjamini-Hochberg procedure (44).

## 3 Results

### 3.1 Sample characteristics

**Figure 2:**
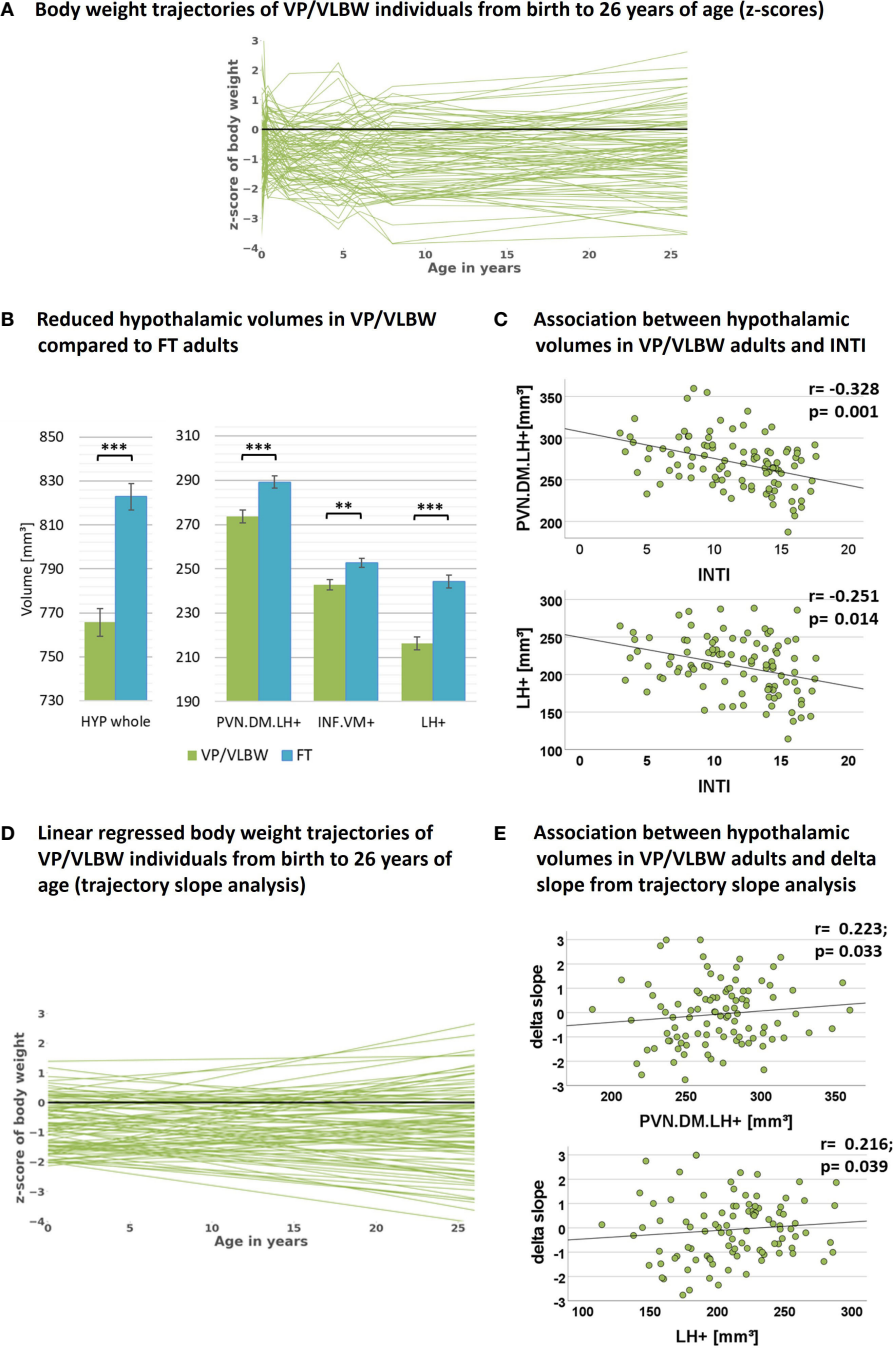
Hypothalamic volumes, prematurity, and body weight trajectories: **(A)** Individual body weight trajectories in the VP/VLBW cohort representing z-scores of body weight at birth and age 5, 20, 56 months, 6, 8 and 26 years. **(B)** Reduced whole hypothalamus and subunit volumes in the VP/VLBW compared to the FT cohort. Marginal means of whole hypothalamus and its subunits are given in mm³ and are shown as bar plots; error bars indicate SE. Group differences were assessed using a general linear model (fixed factor: prematurity at birth, covariates of no interest: sex, scanner, TIV). Group difference significance is marked by asterisks (**: p-FDR < 0.01; *** p-FDR < 0.001). **(C)** Associations between hypothalamic volumes and variables of preterm birth in the VP/VLBW cohort verified by partial correlation analysis. Scatterplots show exemplary relationships between hypothalamus subunit volumes and INTI (for further results see **Supplementary Table S7**). **(D)** Linear regression model of individual body weight trajectories in the VP/VLBW cohort representing body weight development *slopes via* linear regression of individual z-scores of body weight at birth, age 5, 20, 56 months and 6, 8 and 26 years (trajectory long-term slope analysis). **(E)** Association between hypothalamic volumes and long-term delta slope in the VP/VLBW sample. Scatterplots show relationships of hypothalamus subunits with long-term delta slope representing change of body weight z-score from birth until adulthood of linear regressed body weight trajectories. Linear regression lines and regression coefficients of partial regression analysis are added. *Abbreviations:* FDR, false discovery rate correction for multiple comparisons using the Benjamini–Hochberg method; FT, full-term; INTI, intensity of neonatal treatment; SE, standard error; VP/VLBW, very preterm and/or very low birth weight.

**Table 1** and **Figure 2A** show group demographical-clinical, neurocognitive, and body weight-related variables, including longitudinal body weight measurements and their z-scores leading to body weight trajectories of VP/VLBW individuals. 26 individuals of the VP/VLBW cohort fulfilled only the VP criterion (GA< 32 weeks), 21 only the VLBW criterion (BW<1,500g; 32 ≤ GA ≤ 36, GA mean: 33.7 thus still being preterm) and 54 fulfilled both criteria. There were no significant differences between the VP/VLBW and FT group regarding age at scanning (p=0.165), sex (p=0.806), and SES at birth (p=0.770). By design, VP/VLBW subjects had significantly lower GA (p<0.001) and lower BW (p<0.001). VP/VLBW subjects had significantly lower FS-IQ scores in adulthood (p<0.001). Notably, all longitudinal body weight measurements, including adult body weight, were significantly lower in VP/VLBW individuals compared to FT controls.

**Table 1:** Demogaphical-clinical data and body weight variables.

		VP/VLBW			FT			
	Samples	M	SD	Range	M	SD	Range	p-Value
Sex (male/female)	101/110	58/43			65/45			0.806
Age (years)	101/110	26.7	± 0.61	25.7 - 28.3	26.8	± 0.74	25.5 - 28.9	0.165
GA (weeks)	101/110	30.5	± 2.1	25 - 36	39.7	± 1.1	37 - 42	<0.001
INTI, a.u.	100/-	11.6	± 3.8	3 - 18	n.a.	n.a.	n.a.	n.a.
SES^a^, a.u,	101/110	29/44/28		1 - 3	35/49/26		1 - 3	0.770
FS-IQ, a.u.	97/107	94.1	± 12.7	64 - 131	102.6	± 12.0	77 - 130	<0.001
		M	SE	Range	M	SE	Range	p-Value
TIV (cm³)	101/110	1,458	± 11.4	1,435-1,480	1,530	± 10.9	1,508-1,551	<0.001
Birth weight (g)	97/107	1,325	± 39.3	1,248-1,402	3,405	± 37.4	3,331-3,479	<0.001
Body weight, 5m (kg)	90/107	6.56	± 0.09	6.40 - 6.73	7.38	± 0.08	7.23 - 7.53	<0.001
Body weight, 20m (kg)	97/107	9.77	± 0.26	9.25 - 10.3	11.4	± 0.25	10.9 - 11.9	<0.001
Body weight, 56m (kg)	92/104	15.7	± 0.38	14.9 - 16.4	18.2	± 0.36	17.5 - 18.9	<0.001
Body weight, 6y (kg)	84/105	20.1	± 0.32	19.5 - 20.8	21.7	± 0.29	21.2 - 22.3	<0.001
Body weight, 8y (kg)	86/105	25.1	± 0.49	24.1 - 26.1	28.0	± 0.45	27.2 - 28.9	<0.001
Body weight, 26y (kg)	97/107	67.4	± 1.4	64.6 - 70.2	77.2	± 1.3	74.5 - 79.8	<0.001

Statistical comparisons: Sex, SES with χ2 statistics; Age, GA and FS-IQ with two-sample t-tests; TIV at age 26 (covariates: sex, scanner) and all body weight related variables (covariate: sex) with general linear model.

a.u., arbitrary units; FS-IQ, full-scale intelligence quotient; FT, full-term; GA, gestational age; INTI, intensity of neonatal treatment index; Samples, cohort sample size (VP/VLBW and FT); SD, standard deviation; SE, standard error; SES, socioeconomic status at birth; TIV, total intracranial volume; VP/VLBW, very preterm and/or very low birth weight.

a 1 = upper class; 2 = middle class; 3 = lower class. n.a., not applicable.

### 3.2 Lower hypothalamic volumes in VP/VLBW adults linking with body weight trajectories

To investigate whether hypothalamic volumes, including subunits involved in body weight control, are altered after preterm birth, we applied a general linear model approach controlling for sex, scanner, and TIV. We found significantly lower volumes in whole hypothalamus, PVN.DM.LH^+^, INF.VM^+^ and LH^+^ (all p-values <0.002) in VP/VLBW versus FT adults (**Figure 2B**; **Table 2A**).

**Table 2:** Group- and subgroup-based comparisons of hypothalamic volumes.

A. Hypothalamic volumes (VP/VLBW & FT cohort)									
	VP/VLBW (n=101)				FT (n= 110)				
	**M**	**SE**	**95% CI**		**M**	**SE**	**95% CI**		**p-value**
HYP whole (mm³)	765.7	6.3	753.4	778.0	822.9	6.0	811.1	834.6	<0.001***
PVN.DM.LH^+^ (mm³)	273.7	2.9	268.0	279.4	289.1	2.7	283.7	294.5	<0.001***
INF.VM^+^ (mm³)	242.8	2.2	238.4	247.1	252.6	2.1	248.5	256.8	0.002**
LH^+^ (mm³)	216.3	3.0	210.5	222.1	244.1	2.8	238.6	249.7	<0.001***
B. Hypothalamic volumes (VP/VLBW cohort)									
	SGA VP/VLBW (n= 30)				AGA/LGA VP/VLBW (n= 67)				
	M	SE	95% CI		M	SE	95% CI		p-value
HYP whole (mm³)	784.4	12.3	759.9	808.9	743.7	8.2	727.4	759.9	0.008**
PVN.DM.LH^+^ (mm³)	277.1	5.0	267.3	287.0	268.5	3.3	262.0	275.0	0.154
INF.VM^+^ (mm³)	248.0	4.2	239.6	256.4	237.7	2.8	232.2	243.3	0.047**^+^**
LH^+^ (mm³)	225.5	5.8	213.9	237.1	205.4	3.9	197.7	213.1	0.006**
C. Hypothalamic volumes (SGA VP/VLBW cohort)									
	SGA VP/VLBW no catch-up (n=10)				SGA VP/VLBW catch-up (n=20)				
	M	SE	95% CI		M	SE	95% CI		p-value
HYP whole (mm³)	785.1	17.0	749.8	820.3	767.1	11.9	742.5	791.6	0.403
PVN.DM.LH^+^ (mm³)	271.2	7.4	256.0	286.5	275.3	5.1	264.7	285.9	0.659
INF.VM^+^ (mm³)	255.3	6.1	242.7	267.9	238.9	4.3	230.1	247.8	0.042**^+^**
LH^+^ (mm³)	228.2	8.9	209.8	246.5	218.3	6.2	205.5	231.1	0.379

Marginal mean values of whole hypothalamus and its subunits are given in mm³. General linear models **(A)** with prematurity status at birth as fixed factor, **(B)** with z-scores of birth weight below (=SGA)/above (=AGA/LGA) 10th percentile in VP/VLBW cohort as fixed factor, **(C)** with endpoint catch-up growth status in SGA subgroup as fixed factor and scanner, sex, and TIV as covariates of no interest. Group difference significance is marked by asterisks (+: p < 0.05; *: p-FDR < 0.05; **: p-FDR < 0.01; *** p-FDR < 0.001).

AGA/LGA, appropriate for gestational age/large for gestational age; CI, confidence interval; FDR, false discovery rate correction for multiple comparisons using the Benjamini–Hochberg method; FT, full-term; M, mean; SE, standard error; SGA, small for gestational age; VP/VLBW, very preterm and/or very low birth weight.

As VP/VLBW subjects had enlarged lateral ventricles (ca. 25% of VP/VLBW, for detailed analysis see (45)), TIV as control variable might even underestimate the group differences in hypothalamus tissue volumes between the two groups. Therefore, we also adjusted for total gray matter volume instead of TIV, which resulted in p-values <0.001 for hypothalamus volume differences between VP/VLBW and FT adults (see **Supplementary Table S4**), suggesting that lower hypothalamus volumes were not influenced by ventricle volume changes.

Additionally, we performed exploratory analyses of sexual dimorphism and lateralization of hypothalamic volumes for the VP/VLBW and FT cohort. We used general linear models with fixed factor sex or sidedness of hypothalamic hemisphere, respectively, and covariates scanner and TIV (and sex for test of lateralization). No significant sex differences could be detected, although whole hypothalamic volumes of full-term born adults had a trend to be larger in male individuals (comp. **Supplementary Table S5**). In both cohorts, whole hypothalamus volume and all considered subunits (except for the LH^+^ subunit) showed a significant lateralization of hypothalamic volumes to the left (**Supplement Table S6**).

To test whether hypothalamic volume reductions in VP/VLBW adults are indeed related to preterm birth, namely preterm birth-related variables GA and INTI, we performed partial correlation analyses corrected for sex, scanner and TIV. We found significant positive associations between GA and volumes of whole hypothalamus (r=0.265, p=0.009), INF.VM^+^ (r=0.247, p=0.016), and LH^+^ (r=0.236, p=0.021) as well as significant negative associations between INTI and volumes of whole hypothalamus (r=-0.296, p=0.004), PVN.DM.LH^+^ (r=-0.328, p=0.001), and LH^+^ (r=-0.251, p=0.014) (**Figure 2C** for INTI only, **Supplementary Table S7**), suggesting that lower volumes are indeed related with preterm birth.

To test whether hypothalamic volume reductions were related with adult body weight, we applied with respect to adult body weight both partial correlation (corrected for sex, scanner and TIV) and mediation analyses. Concerning correlation analysis, we found a positive correlation between PVN.DM.LH^+^ volume and adult body weight (r=0.214, p=0.041; **Supplementary Table S8**). For mediation analysis (**Supplementary Figure S3**) the three body weight control-related hypothalamus subunits, PVN.DM.LH^+^, INF.VM^+^ and LH^+^, were taken as parallel potential mediators of the relation between INTI or GA, respectively, and adult body weight. We found that the PVN.DM.LH^+^ subunit served as a selective mediator of the association between INTI and adult body weight for VP/VLBW adults. These results indicate the relevance of the PVN.DM.LH^+^ subunit for adult body weight after preterm birth.

To test whether VP/VLBW body weight trajectories were linked with adult hypothalamic volumes, we performed a trajectory slope analysis in the VP/VLBW group. We identified body weight development slopes of individuals by adapting a linear regression model to the measured longitudinal body weight z-scores (**Figure 2D**). Subsequently, change of body weight z-scores from birth to adulthood of regressed body weight trajectories (long-term delta slope) was used for partial correlation analysis with hypothalamic volumes (**Figure 2E**; **Supplementary Table S9**). Whole hypothalamus volume (r=0.238, p=0.022), PVN.DM.LH^+^ (r=0.223, p=0.033), and LH^+^ (r=0.216, p=0.039) were positively correlated with body weight development trajectory long-term slopes, suggesting that body weight trajectories link with adult hypothalamic volumes in prematurity.

### 3.3 Subgroup analysis: adults with long-term catch-up growth

**Figure 3:**
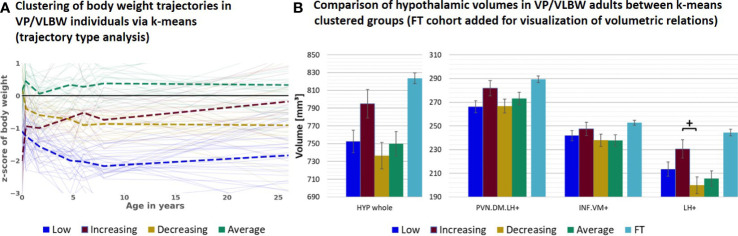
Hypothalamic volumes and body weight trajectories into adulthood – long-term catch-up growth subgroup: **(A)** Clustering of body weight trajectories in the VP/VLBW cohort *via* k-means algorithm (four cluster predefined); k-means grouping: “low” (dark blue), “increasing” (red), “decreasing” (yellow) and “average” (green) (trajectory type analysis). **(B)** Relatively largest HYP volumes of “increasing” group within VP/VLBW cohort. Marginal means of HYP volumes are given in mm³ for k-means clustered groups of VP/VLBW cohort and are shown as bar plots; subunit volumes of FT group (light blue) are added for evaluating volume changes in subgroups relative to FT subjects; error bars indicate SE. Group differences were assessed using a general linear model (fixed factor: four-part variable considering k-means clustered VP/VLBW group differentiation, covariates of no interest: sex, scanner, TIV). Group difference significance is marked by asterisks (+: p < 0.05). FDR, false discovery rate correction for multiple comparisons using the Benjamini–Hochberg method; FT, full-term; SE, standard error; VP/VLBW, very preterm and/or very low birth weight.

Next, to study further special subgroups for associations between hypothalamic volumes and body weight trajectories, we focused now on all VP/VLBW individuals who showed long-term catch-up growth (**Figure 3**). To identify these individuals, we performed a body weight trajectory cluster analysis using a k-means algorithm approach. Based on optimal cluster size of n=4, which was determined as a trade-off between maximizing explained variance score *via* Python (compare **Supplementary Figure S4**) and generating functionally explainable body weight trajectory cluster, we found four distinct types (i.e., cluster) of body weight development (**Figure 3A**) which we called “low” (i.e., individuals with stable relative low body weight; n=32 individuals), “increasing” (i.e., individuals with increasing - from relative low to almost normal - body weight; n=18), “decreasing” (i.e., individuals with decreasing - from almost normal to relative low - body weight; n=21) and “average” (i.e., individuals with stable normal body weight; n=26) weight trajectory. Particularly, the “increasing” trajectory group (red color) represented individuals who started with lowest birth weight z-scores (-2.026) in comparison to all other VP/VLBW clustered groups, but increased their body weight z-scores during development into adulthood to almost normal; we used this group as a proxy for the long-term catch-up growth group. Using a general linear model with these four clustered groups as fixed factor, we found that the “increasing” group had relatively largest hypothalamic volumes within the VP/VLBW cohort (see **Figure 3B**), with a volumetric difference most distinct for the LH^+^ subunit (p=0.024), when compared with the “decreasing” group (yellow color). By adding FT cohort as fixed factor into a further general linear model, we found that the “increasing” group’s hypothalamic volumes even were not different to those of the FT cohort, particularly not for whole hypothalamus, PVN.DM.LH^+^ and LH^+^ subunits, while for the other groups (i.e., low, decreasing, and average group) whole hypothalamus and LH^+^ volumes were smaller compared to FT (see **Supplementary Figure S5**).

We additionally performed an exploratory analysis on the effect of early body weight development. Therefore, we focused on potential volumetric differences of adult hypothalamic volumes comparing VP/VLBW individuals with early catch-up or early catch-down growth in body weight within the first two years of life or stable conditions. No significant differences in whole hypothalamus and respective subunits were detected among these groups, although volumes had a trend to be larger particularly for the group of “early catch-up” in comparison to the group of “early catch-down” (compare **Supplement Table S10**). Partial correlation analysis, restricted to the VP/VLBW group and corrected for sex, scanner and TIV, was used to investigate the associations between adult hypothalamic volumes and “short-term delta slope”. In line with results on volumetric level this correlation analysis provided significant positive correlation factors for whole hypothalamus and the respective subunits (see **Supplement Table S11**). Additionally, there was a significant correlation between “short-term delta slope” and birth weight z-scores (r=-0.726; p<0.001) and between “short-term delta slope” and “long-term delta slope” (r=306; p=0.002). 10 out of the 19 VP/VLBW individuals showing early catch-up were also part of the 18 individuals with “increasing” long-term catch-up growth, 24 out of the 33 VP/VLBW individuals showing early catch-down were also part of the 53 individuals with “decreasing” or “low” long-term body weight development.

### 3.4 Subgroup analysis: adults born with very low birth weight

**Figure 4:**
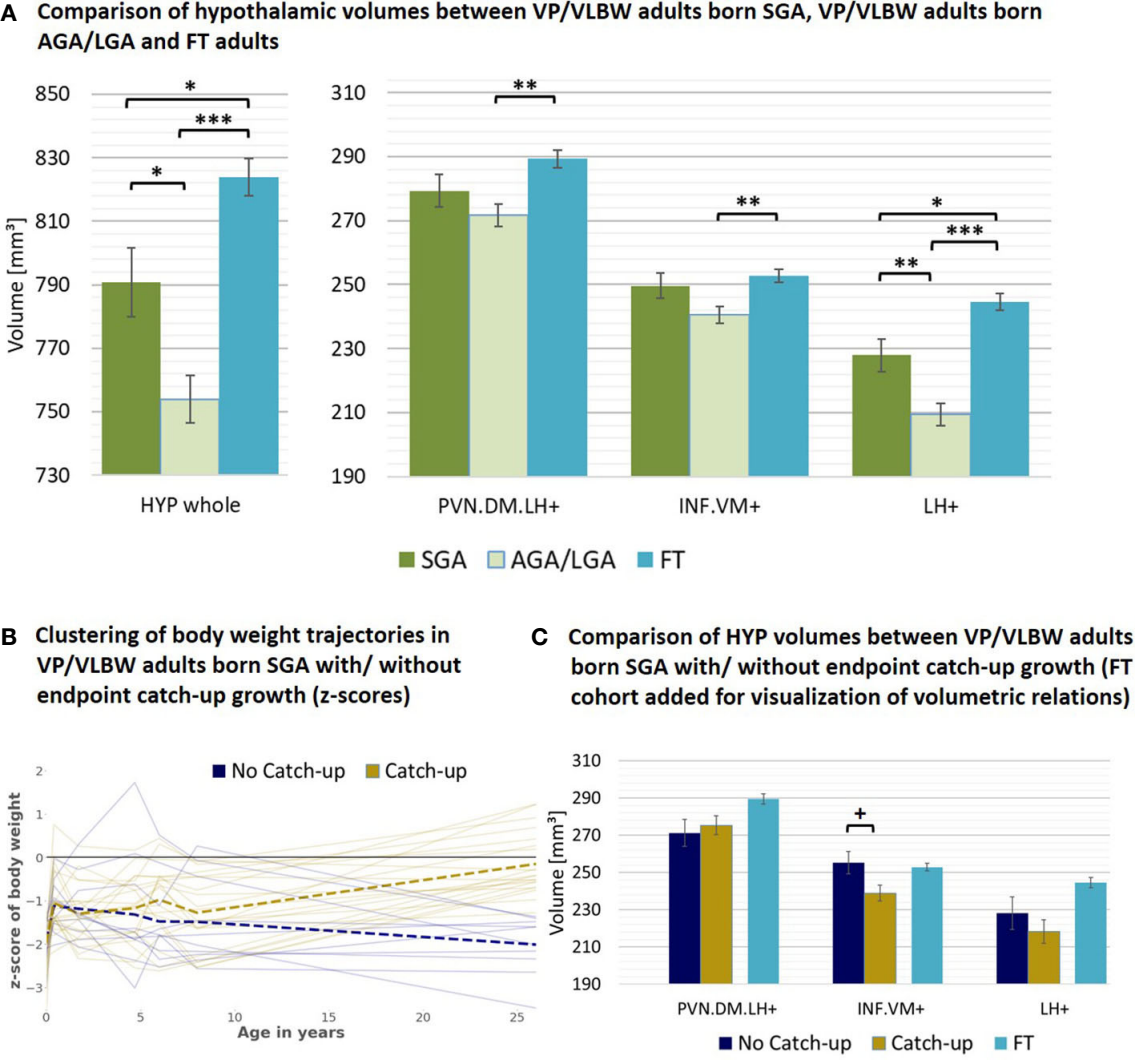
Hypothalamic volumes and birth weight – small for gestational age subgroup: **(A)** Relatively increased HYP volumes of VP/VLBW adults born SGA compared to those VP/VLBW adults born AGA/LGA. Marginal means of HYP volumes are given in mm³ and are shown as bar plots; error bars indicate SE. Group differences were assessed using a general linear model (fixed factor: SGA, AGA/LGA and FT group differentiation status, covariates of no interest: sex, scanner, TIV). Group difference significance is marked by asterisks (+: p < 0.05; *: p-FDR < 0.05; **: p-FDR < 0.01; *** p-FDR < 0.001). **(B)** Individual body weight trajectories in the VP/VLBW SGA cohort. Endpoint catch-up growth was assessed successful if SGA individuals exceeded 10th percentile of adult body weight z-score (yellow trajectories) and failed if not (blue trajectories). Dashed bold lines represent mean body weights of both cohorts at each point of time. **(C)** Hypothalamic volumes in endpoint catch-up and no endpoint catch-up SGA group. Marginal means of HYP subunits are given in mm³ for the SGA cohort comparing endpoint catch-up and no endpoint catch-up growth and are shown as bar plots; subunit volumes of FT group are added for evaluating volume differences in subgroups relative to FT subjects; error bars indicate SE. Group differences were assessed using a general linear model (fixed factor: endpoint catch-up growth status, covariates of no interest: sex, scanner, TIV). AGA/LGA, appropriate for gestational age/large for gestational age; FDR, false discovery rate correction for multiple comparisons using the Benjamini–Hochberg method; FT, full-term; SE, standard error; SGA, small for gestational age; VP/VLBW, very preterm and/or very low birth weight.

As a next subgroup, we focused on VP/VLBW individuals of lower birth weight, particularly individuals born SGA. To start with, we tested whether adult hypothalamic volumes were related to birth weight in the VP/VLBW group using a partial correlation approach corrected for sex, scanner, and TIV. We found, unexpectedly, significant *negative* correlations, namely for the whole hypothalamus (r=-0.262, p=0.012), INF.VM^+^ (r=-0.231, p=0.027), and LH^+^ (r=-0.273; p=0.009) (**Supplementary Table S12**). Correspondingly, when comparing VP/VLBW individuals born SGA (n=30) and those born AGA/LGA (n=67), we found larger volumes for the SGA group, significant for whole hypothalamus, LH^+^ and INF.VM^+^ (**Table 2B**). To test whether SGA hypothalamic volumes were comparable with those of FT controls, we added FT controls into the general linear model (**Figure 4A**; **Supplementary Table S13**) and found indeed that INF.VM^+^ volumes of the SGA group did not differ significantly from those of the FT group.

Then, we studied body weight trajectories of VP/VLBW adults born SGA. We asked whether - within this SGA subgroup - hypothalamic volumes differed with respect to individuals’ body weight trajectory after birth, namely for the subgroups of SGA with ‘successful’ (n=20) or ‘failed’ (n=10) endpoint catch-up growth, defined as *adult* body weight z-score being above or below the 10th percentile. Remarkably, the SGA group of failed endpoint catch-up growth had relatively larger volumes than their successful catch-up counterpart for INF.VM^+^ (p<0.05), with volumes in the range of that in the FT group (**Figures 4B, C**; **Table 2C**).

## 4 Discussion

Using T1-weighted MRI, deep-learning-based hypothalamus subunit segmentation, and longitudinal body weight assessment into adulthood, we observed reduced volumes of whole hypothalamus and hypothalamus subunits relevant for body weight control in VP/VLBW adults, with volume reductions being distinctively associated with long-term body weight trajectories after preterm birth.

### 4.1 Volumes of body weight control-related hypothalamus subunits are reduced in VP/VLBW adults and link with body weight trajectories into adulthood

Volumes of whole hypothalamus and those of body weight control-related subunits PVN.DM.LH^+^, INF.VM^+^ and LH^+^ were reduced in VP/VLBW adults compared to FT controls (**Figure 2B**). This result was not confounded by sex, scanner, and differences in TIV, as we controlled for these factors. Control of TIV suggests relatively stronger hypothalamic, as compared to general brain volume reduction after preterm birth. Volume reductions of VP/VLBW adults were related to GA and INTI, suggesting that hypothalamic volume reductions were indeed linked with preterm birth (**Figure 2C**). Hypothalamic volume reductions are in line with long-term volume reductions in other subcortical gray matter structures after preterm birth, such as cholinergic basal forebrain, thalamus or basal ganglia nuclei (46–49). Furthermore, hypothalamic volume reductions were associated with both altered adult body weight (**Supplementary Figure S3**, **Table S8**) and body weight trajectories in general, namely trajectory long-term slopes (**Figure 2E**, **Supplementary Table S9**), indicating that hypothalamic volume reductions are relevant for impaired long-term body weight gain of prematurity.

Regarding potential microscopic causes of hypothalamic volume reductions, we speculate that canonical hypoxic-ischemic events-induced pathways of both primary impairment of transient cells, namely pre-oligodendrocytes (Pre-OLs) and subplate neurons, and secondary activation of reactive neuroinflammatory cells, such as microglia or astrocytes, might be relevant (50–53). In particular, pre-OLs are responsible for myelination and axonal maturation of connections in general (51), suggesting that pre-OL development impairments are relevant for alterations of hypothalamic nuclei, which are characterized by a high level of long-range connections. We also cannot exclude direct hypoxic-ischemic damage on hypothalamic cells. Beyond perinatal brain injuries, it has been further suggested that additional factors, such as malnutrition and maternal, fetal or postnatal stress, do not only alter feeding behavior and body weight gain (54), but also hypothalamic development of preterm-born infants (24, 51, 55–57).

Based on our exploratory analyses, we could not detect significant sex differences in hypothalamic volumes in both the VP/VLBW and FT cohorts, although according to animal studies several hypothalamic nuclei are known to be sexually dimorphic with regards to regulatory functions and morphometry (21, 58–60). Remarkably, we found a trend to significance of larger whole hypothalamus volumes in full-term born males, being in line with a recent study detecting indeed larger volumes of the whole hypothalamus in adult males (28). We suggest that our MRI-based approach on hypothalamus structure might not be sensitive (enough) to sex differences.

Additionally, we could confirm human imaging studies (28, 61) detecting a larger left than right whole hypothalamus volume. In our case this link was also valid for the PVN.DM.LH^+^ and the INF.VM^+^, but not the LH^+^ subunit, namely for the FT as well as for the VP/VLBW cohort. For literature describing the asymmetrically representation of certain hypothalamic functions and transmitters compare (e.g., 21, 62, 63). In total, the last results further confirm the representative character of our sample with respect to hypothalamic volumes.

### 4.2 VP/VLBW adults with long-term body weight catch-up, relatively larger hypothalamus nuclei, and the lateral hypothalamus

By the use of k-means clustering of body weight trajectories (trajectory type analysis), we identified a special group of VP/VLBW individuals with long-term catch-up growth (“increasing” group; compare **Figure 3**). Remarkably, hypothalamic nuclei volumes of this VP/VLBW long-term catch-up group were not different to those of the FT group, while the other three VP/VLBW groups had lower hypothalamic volumes for whole hypothalamus and LH^+^ (**Supplementary Figure S5**); additionally, LH^+^ volumes of the long-term catch-up group were relatively larger than those of the other VP/VLBW trajectory groups (**Figure 3B**). Therefore, relatively larger hypothalamic volumes, particularly for subunits including the lateral hypothalamus, seem to be associated with more favorable long-term body weight trajectories in VP/VLBW. This observation is in line with findings that volumetric increases of hypothalamus gray matter and hence its nuclei are linked with altered hypothalamic regulation of body weight (64). Furthermore, it matches results in humans that revealed a positive association of general brain growth with optimized early nutrition (65) and body growth (66).

Based on our exploratory analysis on early catch-up, we could show that VP/VLBW individuals with relatively low birth weight and an early catch-up in the first two years of life are linked to the respective positive long-term trend of body weight development until adulthood and are more probable in expressing relatively larger adult hypothalamic volumes than those of e.g. early catch-down growth. Taken together, those relations match our observations on hypothalamic volumes for the long-term “increasing” catch-up group. Unfortunately, due to missing MRI data at birth, we cannot decide whether relatively larger hypothalamus volumes, especially of the lateral hypothalamus, are a result of a relatively increasing hypothalamus during development (i.e., volumetric hypothalamus catch-up) or of already at birth relatively larger hypothalamus volumes. Future studies of the infant hypothalamus are necessary to test this suggestions, with implications for both optimized nutrition (67) and neuroprotective/neurorestorative interventions (51) to prevent potential aberrant hypothalamic development.

### 4.3 VP/VLBW adults born SGA have larger hypothalamic volumes; SGA without endpoint catch-up growth and infundibular-ventromedial nuclei

We unexpectedly found negative correlations between adult hypothalamic volumes and birth weight in VP/VLBW adults (**Supplementary Table S12**). Correspondingly, VP/VLBW adults born SGA have relatively larger adult hypothalamic volumes than their AGA/LGA counterparts (**Figure 4A**).

In preterm-born babies being SGA, similar results have been reported for the pituitary structure, with its height being increased (68, 69). Contrary to that, several studies have linked preterm-born infants being SGA or those with intrauterine growth restriction (IUGR) with reduced cortical (70, 71) and subcortical gray matter volumes such as hippocampus, basal ganglia or thalamus nuclei (72, 73).

Therefore, to explain relatively increased hypothalamic volumes in VP/VLBW adults born SGA, two non-exclusive scenarios are conceivable: First, hypothalamic volume increases might already be present in infancy or even at birth (as in the case of the pituitary gland) and/or second, they might develop postnatally over time. The first hypothesis is supported by observed hyperactivity of the human hypothalamic-pituitary axis in preterm-born SGA neonates due to hormonal changes (e.g., growth-hormone (GH), IGF-1) (11, 68, 74), suggesting compensatory enlargement of the hypothalamus. It is also supported by animal studies showing higher cell counts or relative volumes of certain body weight control-related nuclei in a (non-prematurity) SGA model, potentially due to intrauterine neuroendocrine changes and epigenetic modifications (75, 76).

The second hypothesis is supported by studies that demonstrated higher vulnerability of gray matter for adverse effects of prematurity on SGA or IUGR infants in comparison to AGA (51, 77), suggesting a certain degree of perinatal hypothalamic volume reduction. In this case, the later increased hypothalamic volumes of VP/VLBW born SGA in adulthood could be explained by an altered volumetric hypothalamus development from birth into adulthood (24). Notably, long-term hypothalamic development can be affected by prolonged neurogenesis e.g., based on special neural stem cells called tanycytes, which are also supposed to regulate food intake and energy expenditure (78–80). These cells are located both along the lateral walls near to body weight control-related nuclei and at the floor of the third ventricle, namely nearby the INF.VM^+^ subunit. Their neurogenesis is controlled e.g., by neurotrophic factors like brain-derived neurotrophic factor (BDNF) (81), or nutrition and the endocrine system, including metabolic hormones such as leptin, insulin, ghrelin, and IGF-1 (82, 83). Concentrations of these hormones and molecules are altered in humans after preterm birth (15, 74, 84), specifically for SGA-born individuals (85–87). Besides prematurity and weight status at birth, body weight changes during development until adulthood are linked with altered ghrelin and leptin levels in humans (88, 89). Both hormones affect infundibular and ventromedial nuclei (as parts of INF.VM^+^), main sites of tanycyte neurogenesis (79). This might contribute to our findings of VP/VLBW adults born SGA *without* endpoint catch-up growth having relatively larger hypothalamic volumes, particularly for the INF.VM^+^ subunit, than those with endpoint catch-up growth (**Figures 4B, C**). This result suggests that for the SGA subgroup different hypothalamic nuclei might develop distinctively with respect to body weight development.

## 5 Strengths and limitations

Strengths of our study are the large sample size, enhancing power and generalizability of our findings. Homogenous mean across VP/VLBW and FT groups excludes confounding age effects. Notably, our results should be viewed as conservative estimates of the true group differences including hypothalamic volumes, as our sample is biased towards VP/VLBW adults with less severe neonatal complications and thus adult impairments. VP/VLBW adults with more neonatal complications or functional impairments had an increased probability not to participate in the study due to MRI exclusion criteria or to reject MRI screening. One should note that – beyond exclusion criteria – lack of motivation was the main reason for rejecting MRI and this reason was equally distributed across groups. One should note further that the sample was still representative of the full Bavarian Longitudinal Study cohort in terms of GA and BW distribution (36).

For calculation of body weight z-scores at distinct timepoints along the observed time frame, three different reference populations have been used. Though all three databases are large and represent data from the same country, z-score values may be biased due to relative differences in the reference populations’ body weight means and standard deviations at a particular timepoint.

For trajectory slope analysis (**Figure 2D**) - on first approximation - a linear body weight development model was preferred over a nonlinear one, despite the existence of nonlinearities in the body weight trajectories (**Figure 2A**). Although our sample size is large, future studies based on even larger sample sizes and more frequent body weight mapping along the age trajectory are necessary for nonlinear body weight development modelling

MRI brain scans were only available at age 26, whereas a longitudinal measurement of hypothalamic volumes during development would be required to address several relevant issues, such as separating cause from consequence regarding altered hypothalamic structure or revealing hypothalamic structure interdependency with long-term body weight gain.

This long-term body weight gain and hence its trajectories are – as mentioned – additionally influenced by many factors such as of maternal, genetic, fetal, environmental, nutritional, stress- or diseases related, endocrine, socioeconomic, and neurodevelopmental origin. Future studies are needed to include these factors, as within this study we were primarily interested in the general relationship between preterm birth, hypothalamus, and body weight gain.

A final limitation of our study is the subunit-focused parcellation methodology of the hypothalamus segmentation algorithm by Billot et al. (33). As their subunit definition is bound to anatomical landmarks, therefore aggregating several hypothalamic nuclei per subunit, it is challenging to allocate differences in subunit volumes to distinct body weight control-related hypothalamic nucleus volumes and hence their functional implications.

## 6 Conclusion and outlook

Our results demonstrate an association of preterm birth with body weight control-related hypothalamus subunits that link, in turn, with long-term body weight gain after preterm birth.

The presented approach suggests a potential of hypothalamus imaging for both weight development prognosis and potential treatment monitoring. It might also be translated to other disorders of impaired body weight development, from obesity to anorexia nervosa and treatment-induced body weight changes.

## Data availability statement

The datasets presented in this study can be found in online repositories. The names of the repository/repositories and accession number(s) can be found below: Hypothalamus volumes and metadata of segmented individuals was uploaded *via* Dryad (https://datadryad.org/stash/share/qUpAwamRz0qThUhbBdqmtr5nTQqAYSdqR4uSDTfOxKs; doi:10.5061/dryad.vdncjsxwp). More data that support the findings of this study are available for interested researchers from the corresponding author upon reasonable request. The code used to generate and analyze the data was published by FreeSurfer (http://surfer.nmr.mgh.harvard.edu/) and can be found *via* https://surfer.nmr.mgh.harvard.edu/fswiki/HypothalamicSubunits.

## Ethics statement

The studies involving human participants were reviewed and approved by Ethics committee of the Klinikum rechts der Isar and the University Hospital Bonn. Written informed consent to participate in this study was provided by the participants’ legal guardian/next of kin.

## Author contributions

TR: Data curation; Software; Formal analysis; Validation; Investigation; Visualization; Methodology; Writing - original draft; Writing – review and editing. BS-K: Writing - review and editing. AM: Writing - review and editing. RE: Resources; Writing – review and editing. MD: Writing - review and editing. HB: Writing – review and editing. ER-F: Writing - review and editing. JP: Writing - review and editing. CZ: Writing - review and editing. PB: Funding acquisition; Writing - review and editing. DW: Resources; Funding acquisition; Writing - review and editing. CS: Conceptualization; Resources; Supervision; Funding acquisition; Methodology; Writing - original draft; Project administration; Writing - review and editing. DH: Conceptualization; Resources; Supervision; Funding acquisition; Methodology; Writing - original draft; Project administration; Writing - review and editing. All authors contributed to the article and approved the submitted version.
