# Supplementary material for: Lower hypothalamus subunit volumes link with impaired long-term body weight gain after preterm birth
Source: Front Endocrinol (Lausanne). 2022 Dec 15;13:1057566. doi: 10.3389/fendo.2022.1057566 (PMC9797519; doi:10.3389/fendo.2022.1057566)
Supplement: Supplementary file 1 [file DataSheet_1.docx]

Supplementary Material

(by Tobias Ruzok et al.)

# Content Table

[1 Content Table 1](#_Toc119855334)

[2 Supplementary Materials and Methods 3](#_Toc119855335)

[2.1 Participants and ‘clinical-qualitative’ brain MRI 3](#_Toc119855336)

[2.2 Gestational age, medical treatment at birth, and family socioeconomic status 3](#_Toc119855337)

[2.3 Body weight development 4](#_Toc119855338)

[2.4 Neurocognitive assessment 5](#_Toc119855339)

[2.5 MRI data acquisition 5](#_Toc119855340)

[2.6 Hypothalamus segmentation 5](#_Toc119855341)

[2.7 Statistical analysis 6](#_Toc119855342)

[3 Supplementary Figures 7](#_Toc119855343)

[3.1 Figure S1: Participants of the Bavarian Longitudinal Study 7](#_Toc119855344)

[3.2 Figure S2: Reliability control analysis of segmented whole HYP and representative
 functional subunit (INF.VM+) volumes via comparison to HYP studies in literature 8](#_Toc119855345)

[3.3 Figure S3: VP/VLBW adults: adult body weight and PVN.DM.LH+ 10](#_Toc119855346)

[3.4 Figure S4: K-means cluster analysis of body weight trajectories: differences in
 explained variance regression score with varying number of predefined cluster 11](#_Toc119855347)

[3.5 Figure S5: Comparison of hypothalamic volumes between k-means clustered
 groups of VP/VLBW adults and adult FT cohort 12](#_Toc119855348)

[4 Supplementary Tables 13](#_Toc119855349)

[4.1 Table S1: Intraventricular hemorrhage 13](#_Toc119855350)

[4.2 Table S2: Description of the variables comprising the INTI 14](#_Toc119855351)

[4.3 Table S3: Hypothalamus parcellation methodologies and segmentation results
 in literature 16](#_Toc119855352)

[4.4 Table S4: Group comparison of hypothalamic volumes with respect to gray
 matter volume (instead of TIV) as covariate of no interest 17](#_Toc119855353)

[4.5 Table S5: Group comparison of hypothalamic volumes with regards to sex
 differences 18](#_Toc119855354)

[4.6 Table S6: Group comparison of hypothalamic volumes with regards to
 hypothalamic hemisphere 19](#_Toc119855355)

[4.7 Table S7: Relationship between hypothalamic volumes in VP/VLBW adults and
 variables of preterm birth 20](#_Toc119855356)

[4.8 Table S8: Relationship between hypothalamic volumes in VP/VLBW adults and
 adult body weight 21](#_Toc119855357)

[4.9 Table S9: Relationship between hypothalamic volumes in VP/VLBW adults and
 long-term delta slope of body weight trajectories 22](#_Toc119855358)

[4.10 Table S10: Group comparisons of hypothalamic volumes in the VP/VLBW cohort
 with regards to early development of body weight 23](#_Toc119855359)

[4.11 Table S11: Relationship between hypothalamic volumes in VP/VLBW adults and
 short-term delta slope of body weight trajectories 24](#_Toc119855360)

[4.12 Table S12: Relationship between hypothalamic volumes in VP/VLBW adults and
 birth weight z-scores 25](#_Toc119855361)

[4.13 Table S13: Group comparison of hypothalamic volumes with regards to birth
 weight and status of preterm birth (VP/VLBW adults born SGA vs. VP/VLBW
 adults born AGA/LGA vs. adult FT) 26](#_Toc119855362)

[5 Supplementary References 28](#_Toc119855363)

# Supplementary Materials and Methods

## Participants and ‘clinical-qualitative’ brain MRI

This study assessed a geographically defined whole population sample of neonatal at-risk very preterm and/or very low birth weight (<32 weeks of gestation and/or <1500g) individuals (VP/VLBW) and healthy full-term controls (FT). Sample data were collected as part of the Bavarian Longitudinal Study, of which a more detailed description can be found elsewhere (1, 2). Briefly, all individuals were born between Jan 1^st^, 1985, and March 31^st^, 1986, in a defined region of Southern Bavaria and were followed from birth into adulthood. The VP/VLBW cohort initially consisted of 682 individuals of whom 260 participated in the 26-year follow-up assessment, including measurement of body weight parameters. Of the initial 916 FT controls from the same obstetric hospitals alive at 6 years, 350 were randomly selected within the stratification variables of sex and family socioeconomic status as being comparable to the VP/VLBW cohort. 229 of them participated in the 26-year follow-up assessment. For subsequent brain imaging at age 26, all individuals were screened for MR-related exclusion criteria including (self-reported) claustrophobia, inability to lie still for > 30min, unstable medical conditions (e.g., severe asthma), epilepsy, tinnitus, pregnancy, non-removable MRI-incompatible metal implants, and a history of severe central nervous system (CNS) trauma or disease that would impair further analysis of the data. The most frequent reason not to perform the MRI exam, however, was lack of motivation. The remaining 101 VP/VLBW individuals and 111 FT controls underwent MRI scan.

One should note, although severe brain lesions affecting general brain structure were an exclusion criterion to enable comparability of MRIs across VP/VLBW and FT groups, VP/VLBW individuals had more ‘clinical-qualitative’ MRI changes than FT individuals. For example, VP/VLBW subjects had enlarged lateral ventricles (ca. 25% of VP/VLBW) and more periventricular white matter lesions on a group level (for detailed analysis see (3)). Furthermore, intraventricular hemorrhage in the neonatal period was present in the VP/VLBW group (Supplementary Table S1).

The MRI examinations took place at two sites: The Department of Neuroradiology, Klinikum rechts der Isar, Technical University of Munich (n=146), and the Department of Radiology, University Hospital of Bonn (n=66). For a detailed flowchart of participants through the study see Supplement Fig. S1 and (4).

The study was carried out in accordance with the Declaration of Helsinki and was approved by the local ethics committee of the Klinikum rechts der Isar and the University Hospital Bonn. All study participants gave written informed consent. They received travel expenses and payment for participation.

## Gestational age, medical treatment at birth, and family socioeconomic status

Gestational age (GA) was estimated from maternal reports on the first day of the last menstrual period and from serial ultrasounds during pregnancy. In cases, in which the two measures differed by more than 2 weeks, clinical assessment at birth with the Dubowitz method was applied (5). To estimate medical impairments at birth, Intensity of Neonatal Treatment Index (INTI) was calculated via daily assessments of care level, respiratory support, feeding dependency, and neurological status (mobility, muscle tone, and neurological excitability). Each of these six variables was scored on a 4-point rating scale (0–3) using the method of Casaer and Eggermont (6) (see Supplement Table S2 for a description of the variables). INTI was computed as the mean score of daily ratings during the first 10 days of life or until a stable clinical state was reached (total daily scores <3 for 3 consecutive days), depending on which occurred first, ranging from 0 (best state) to 18 (worst state).
Family socioeconomic status (SES) was assessed through structured parental interviews within 10 days of childbirth. SES was computed as a weighted composite score based on the profession of the self-identified head of each family together with the highest educational qualification held by either parent (7).

## Body weight development

Body weight measurements were undertaken at birth and during follow-up visits at five and 20 months corrected for prematurity, and at 56 months, 6, 8 and 26 years of chronological age by specially trained research nurses. They used predefined protocols with weighing on standard scales in underwear only (8). For body weight development analysis, we transformed body weight measurements from [g]/[kg] into z-scores relative to the exact age of each participant, typically used for the description of longitudinal changes of body weight status (9, 10). In detail, a neonatal reference was applied for calculation of "birth weight for gestational age z-scores" (referred to as "birth weight z-scores" in our study). We used Voigt’s neonatal population database comprising 2.3 million live and still singleton births in Germany from 1995 to 2000 with a GA from 20 to 43 weeks (11, 12), allowing for sex-specific weight percentiles to be calculated. For calculation of body weight z-scores at all other ages a reference was applied comprising percentiles of body weight in children and adolescents including 17.147 males and 17.275 females, aged 0-18 years, evaluated from different regional German studies (13). For ages greater 18 years, the authors additionally integrated percentile data from the German “Mikrozensus” (14) into their reference.

For all analyses focusing on variables regarding body weight development restricted to the VP/VLBW cohort, four individuals were excluded as adult body weight at age 26 was missing (remaining number of individuals of VP/VLBW cohort for body weight development analyses: n=97).

*SGA status and catch-up growth.* “Small for gestational age” (SGA) versus “appropriate/large for gestational age” (AGA/LGA) was determined depending on birth weight z-scores being below (equivalent to z-scores < -1.282) versus above the 10th percentile, respectively (11, 15). Within the SGA subcohort, we defined successful endpoint catch-up growth as an adult body weight z-score at age 26 above the 10th percentile. In doing so, we concentrated on long-term body weight development as opposed to short-term evaluations of catch-up growth within the first two years of life as mostly performed in the literature (16–19). This allowed us to better account for the additional role of long-term body weight gain in metabolic and cardiovascular outcome after preterm birth (20, 21).

*Body weight trajectory analysis.* To analyze the relationship between hypothalamic volumes and body weight trajectories, we performed both trajectory slope and trajectory type analysis using Python version 3.7.10 and especially the “scikit-learn” package, a machine learning focused Python library.

Firstly, for trajectory slope analysis we assessed body weight development restricted to the VP/VLBW group. Linear regression of body weight z-scores (non-interpolated) at birth, ages 5, 20, 56 months and 6, 8 and 26 years was performed to receive regressed body weight trajectories. Partial correlations were used to investigate the associations between change of body weight z-score from birth until adulthood of regressed body weight trajectories (long-term delta slope) and hypothalamic volumes corrected for sex, scanner, and total intracranial volume (TIV; sum of segmented gray and white matter brain volumes and cerebrospinal fluid partitions).

Secondly, for trajectory type analysis, we revealed body weight trajectory types in the VP/VLBW group by a clustering approach. In particular, we clustered body weight trajectories via k-means algorithm in Python (sklearn.cluster.KMeans) after approximating missing data points via linear interpolation (number of missing data points can be deduced from Table 1 with regards to missing samples from maximum n=97 VP/VLBW, n=110 FT at every stage of longitudinal weight analysis). The k-means algorithm is based on minimizing within-cluster sum of squared distances between each data point and the centroids (22–24). The number of times the k-means algorithm was run with different centroid seeds as default initialization was n=100. After trajectory clustering, hypothalamic volumes were compared across cluster using general linear models as described above.

## Neurocognitive assessment

At 26 years of age study participants were assessed using a short version of the German Wechsler Adults Intelligence Scale, Third edition (25). The assessment took place prior to and independent of the MRI scan and was carried out by trained psychologists who were blinded to group membership. Subsequently, an age-normed Full-Scale IQ (FS-IQ) was computed.

## MRI data acquisition

MRI data acquisition (see (26)) was performed at Klinikum rechts der Isar, Technical University of Munich, and Bonn University Hospital on Philips Achieva 3T systems or Philips Ingenia 3T systems using an 8-channel SENSE head coil. Subject distribution among scanners was: Bonn Achieva 3T: 5 VP/VLBW, 11 FT; Bonn Ingenia 3T: 33 VP/VLBW, 17 FT; Munich Achieva 3T: 60 VP/VLBW, 65 FT; Munich Ingenia 3T: 3 VP/VLBW, 17 FT. Across all scanners sequence parameters were kept identical. Scanners were checked regularly to provide optimal scanning conditions and MRI physicists at the University Hospital Bonn and Klinikum rechts der Isar regularly scanned imaging phantoms to ensure within-scanner signal stability over time. Signal-to-noise ratio was not significantly different between scanners (one-way analysis of variance with factor “scanner-ID” [Bonn 1, Bonn 2, Munich 1, Munich 2]; F(3,182)=1.84, p=0.11). A high-resolution T1-weighted 3D magnetization prepared rapid acquisition gradient echo (MPRAGE) sequence (TI=1,300ms, TR=7.7ms, TE=3.9ms, flip angle=15°; 180 sagittal slices, FOV=256×256×180mm, reconstruction matrix=256×256; reconstructed isotropic voxel size=1mm³) was acquired. All images were visually inspected for artefacts. In our study, to account for possible confounds by scanner differences, MRI data analyses included scanner dummy variables as covariates of no interest.

## Hypothalamus segmentation

T1-weighted MRI scans in Nifti-format were processed by using the freely available FreeSurfer image analysis suite (http://surfer.nmr.mgh.harvard.edu/). Particularly, the version of FreeSurfer 7.2 includes a deep convolutional neural network tool of Billot et al. (27) that enables for automated segmentation of the hypothalamus, including subsegment parcellation. Billot and colleagues trained this neural network on thirty-seven T1-weighted, manually labelled, and augmented MRI scans. A state-of-the-art 3D U-net model (28) served as a basis for the architecture of the network. As a result, this hypothalamus delineation approach exceeded former approaches based on multi-atlas segmentation or deep learning-based algorithms (29), and it was comparable to expert intra-rater precision (27). We applied the algorithm to our dataset of T1-weighted MRI scans.

To focus on hypothalamic nuclei involved in body weight control, we mapped subsegments from the segmentation algorithm that contained nuclei relevant for body weight control onto *three so-called subunits of body weight control, namely PVN.DM.LH^+^, INF.VM^+^ and LH^+^*. The PVN.DM.LH^+^ subunit is the aggregate of two subsegments of the segmentation of Billot et al. (27), namely the anterior-superior and superior tubular subsegment (see Fig. 1), and these two subsegments cover the preoptic area, the paraventricular nucleus (PVN), the dorsomedial nucleus (DM), and parts of the lateral hypothalamus (LH), with the latter three being involved in body weight control and therefore defining the name of the subunit. INF.VM^+^ is identical to the inferior tubular subsegment, which comprises - amongst other nuclei - the body weight control-related infundibular (INF) and ventromedial (VM) nuclei. LH^+^ matches the posterior subsegment in Billot et al. (27), including the mammillary bodies, parts of the tuberomammillary nucleus and, critically, of the body weight control-related lateral hypothalamus (LH). The final anterior-inferior subsegment (suprachiasmatic nucleus and parts of the supraoptic nucleus) was excluded from further analysis because it does not contain any body weight control-related nuclei.
All subunits stated in the analysis already consider volumes of bilateral hypothalamus.

## Statistical analysis

Statistical analyses were performed using SPSS version 27 (IBM SPSS Statistics).

*Analyses of group differences and correlation analyses.* Regarding demographical characteristics, group differences between VP/VLBW and FT cohorts were assessed using chi-square tests (sex, SES) and two-sample t-tests (age, GA, FS-IQ). To test whether both hypothalamic volumes and body weights are altered in prematurity, general linear models were used (dependent variable: hypothalamic volumes or body weight, respectively; fixed factor: status of prematurity at birth; covariates: sex and additionally only for hypothalamic volume changes: scanner, TIV). Age was not included as a covariate in our analyses, as VP/VLBW subjects and FT controls were not significantly different in mean age at scanning of 26 years. Partial correlation analysis, restricted to the VP/VLBW group and corrected for sex, scanner and TIV, was used to investigate the associations between hypothalamic volumes and variables of preterm birth i.e., GA and INTI, respectively. Regarding variables related to body weight development (birth weight z-scores and adult weight) similar correlation analyses were applied.

*Mediation analysis.* To assess potential mediation effects of hypothalamic volumes regarding the association of variables of prematurity (i.e., INTI and GA, respectively) with adult body weight, a mediation analysis restricted to the VP/VLBW cohort was performed using the PROCESS toolbox (version 3.5) of SPSS (30). In the mediation model, INTI and GA were entered as causal variables, respectively, adult body weight as the outcome variable, and volumes of PVN.DM.LH^+^, INF.VM^+^ and LH^+^ subunits were introduced simultaneously as potential parallel mediator variables (covariates of no interest: sex, scanner, and TIV). Path coefficients for total effect, direct effect and indirect effect were estimated using (unstandardized) regression coefficients from multiple regression analyses, and statistical significance of the indirect effect was tested using a nonparametric bootstrap approach (with 5000 repetitions) to obtain 95% confidence intervals. We calculated p-values for indirect effects based on 95% confidence intervals, SE and estimated effect as described by Altman and Bland (31).

*Statistical thresholds.* Statistical significance was set at p <0.05; all tests were two-sided. Tests were corrected for multiple comparisons for false discovery rate (FDR) according to the Benjamini-Hochberg procedure (32).

# Supplementary Figures

## Figure S1: Participants of the Bavarian Longitudinal Study


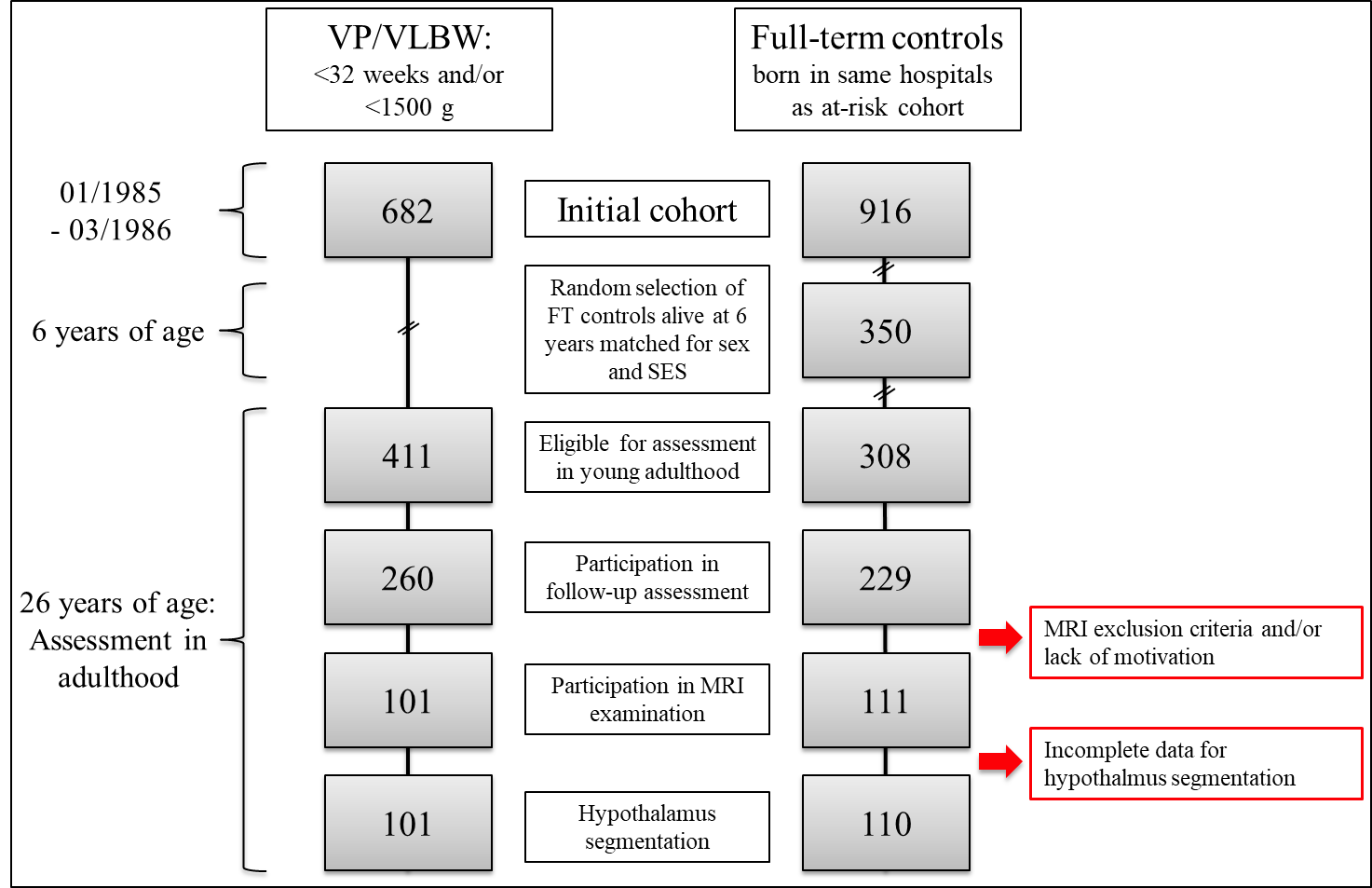


**Figure S1.** Flowchart of the participants of the Bavarian Longitudinal Study.
Abbreviations: MRI, magnetic resonance imaging; SES, socioeconomic status; VP/VLBW, very preterm and/or very low birth weight.

## Figure S2: Reliability control analysis of segmented whole HYP and representative functional subunit (INF.VM+) volumes via comparison to HYP studies in literature


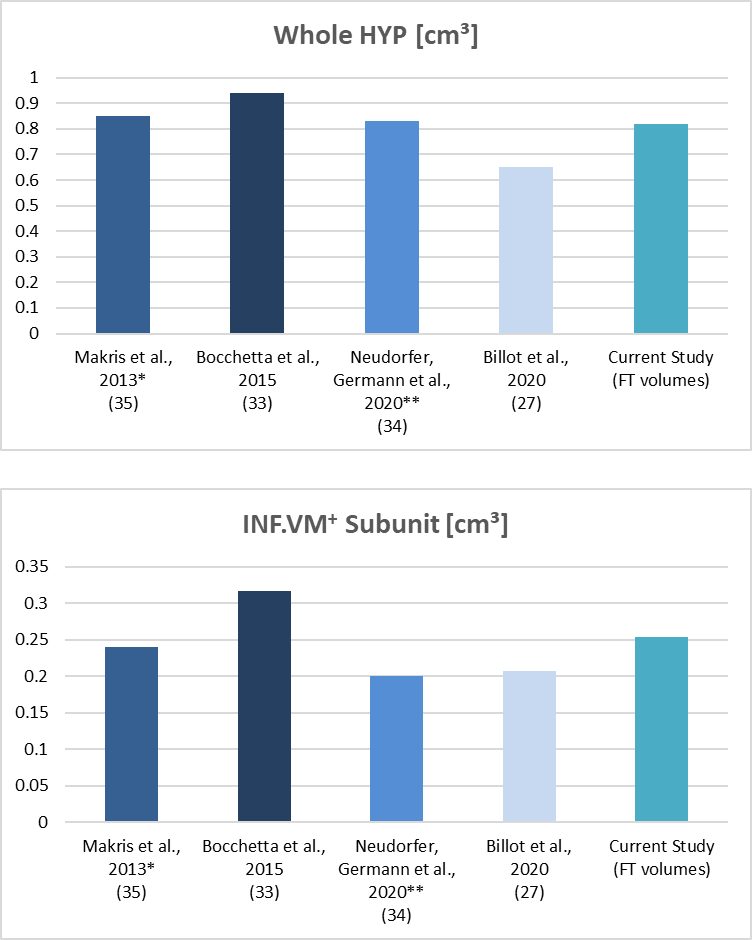


**Figure S2.** Comparison of manually or (semi-)automated segmented hypothalamic volumes (in cm³) in research literature for whole hypothalamus (upper line) and hypothalamus subunits with representative visualization of INV.VM^+^ subunit (lower line). With regards to subunit comparisons, studies were selected which provided similar hypothalamus subunit parcellation methodology. If available, segmentation results of healthy controls were taken for comparison (also compare Supplement Table S3).
Additional remarks: The INF.VM^+^ (inferior tubular) subunit comprises, according to the Billot et al. (27) segmentation algorithm, the infundibular nucleus, the ventromedial nucleus, the lateral tubular nucleus, and parts of the tuberomammillary and the supraoptic nuclei; the lateral tubular nucleus was not assigned to the INF.VM^+^ subunit in Bocchetta et al. (33) and Neudorfer, Germann et al. (34) and the tuberomammillary nucleus not assigned to the INF.VM^+^ subunit in Makris et al. (35) and Bocchetta et al. (33).
*HYP volumes = (male + female volumes)/2 (mean of provided values).
**Summation of single nuclear volumes (as parcellation on nuclear level is available in Neudorfer, Germann et al.) (34).
Abbreviations: FT, full-term; HYP, hypothalamus.

## Figure S3: VP/VLBW adults: adult body weight and PVN.DM.LH+


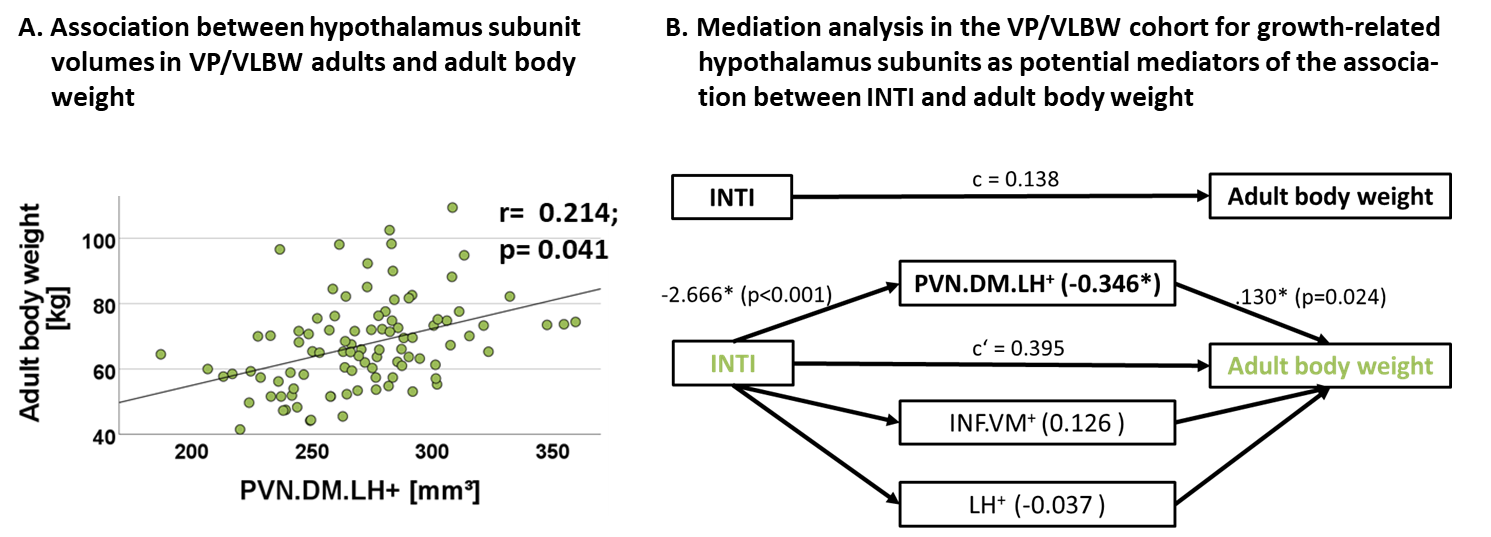


**Figure S3. (A)** Association between hypothalamic volumes and adult body weight in the VP/VLBW cohort (compare also Supplement Table S8). Scatterplot shows relationship between adult body weight and PVN.DM.LH^+^ subunit volumes. Linear regression line and regression coefficient of partial regression analysis are added. **(B)** The PVN.DM.LH^+^ subunit volume mediates the association between medical complications at birth (represented by INTI as causal variable) and adult body weight (in kg). A path diagram is shown in order to illustrate the results of the mediation analyses restricted to the VP/VLBW cohort. The regression model was corrected for sex, scanner and TIV. Body weight related hypothalamus subunits were introduced as potential mediators and the PVN.DM.LH^+^ subunit volume yielded a significant indirect effect (ab= -0.346 ± 0.174; bootstrapped 95% CI: -0.738 to -0.075; p= 0.040) (30). All other subunits did not show significant mediation effects. The figure includes the following standardized regression coefficients: c, the total effect of INTI on adult body weight; c’, the direct effect of INTI on adult body weight when adjusting for the potential mediating variables. Significant regression coefficients (p < 0.05) are marked with an asterisk. For GA as causal variable, we did not find a similar mediation effect.
Abbreviations: INTI, intensity of neonatal treatment; VP/VLBW, very preterm and/or very low birth weight.

## Figure S4: K-means cluster analysis of body weight trajectories: differences in explained variance regression score with varying number of predefined cluster


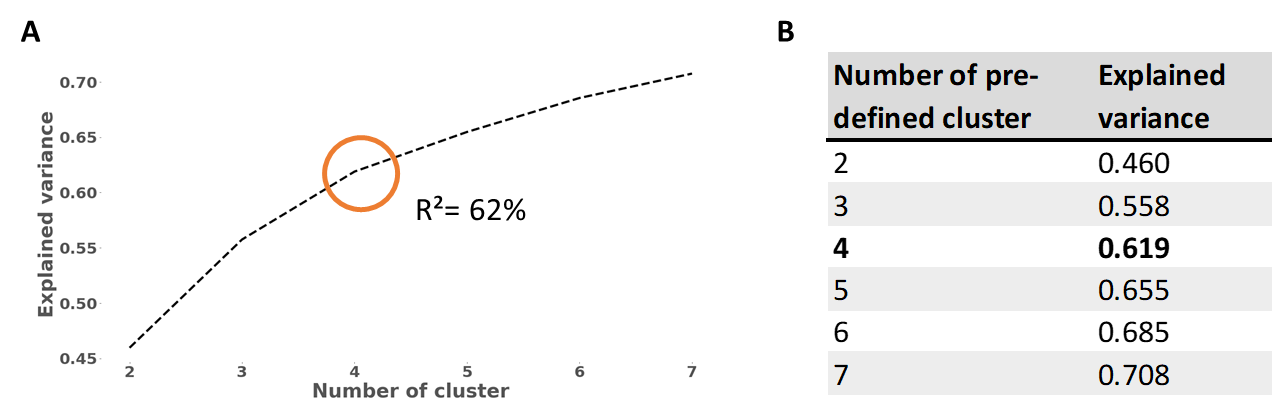
 **Figure S4. *Left.*** Graph of explained variance regression score with varying number of predefined cluster for clustering of body weight trajectories in the VP/VLBW cohort. The explained variance regression score (sklearn.metrics.explained_variance_score) is calculated as *explained_variance(y, y*)= 1- Var(y - y*) / Var(y)*, where *y – y** describes the pointwise calculation of the gap between the individual trajectory under observation (y) and the correspondent clustered trajectory (y*). Var as variance represents the square of the standard deviation. The explained variance regression score is ≤ 1, with 1 being the optimum. ***Right.*** Absolute values of explained variance regression scores depending on number of predefined cluster.
Abbreviations: VP/VLBW, very preterm and/or very low birth weight.

## Figure S5: Comparison of hypothalamic volumes between k-means clustered groups of VP/VLBW adults and adult FT cohort


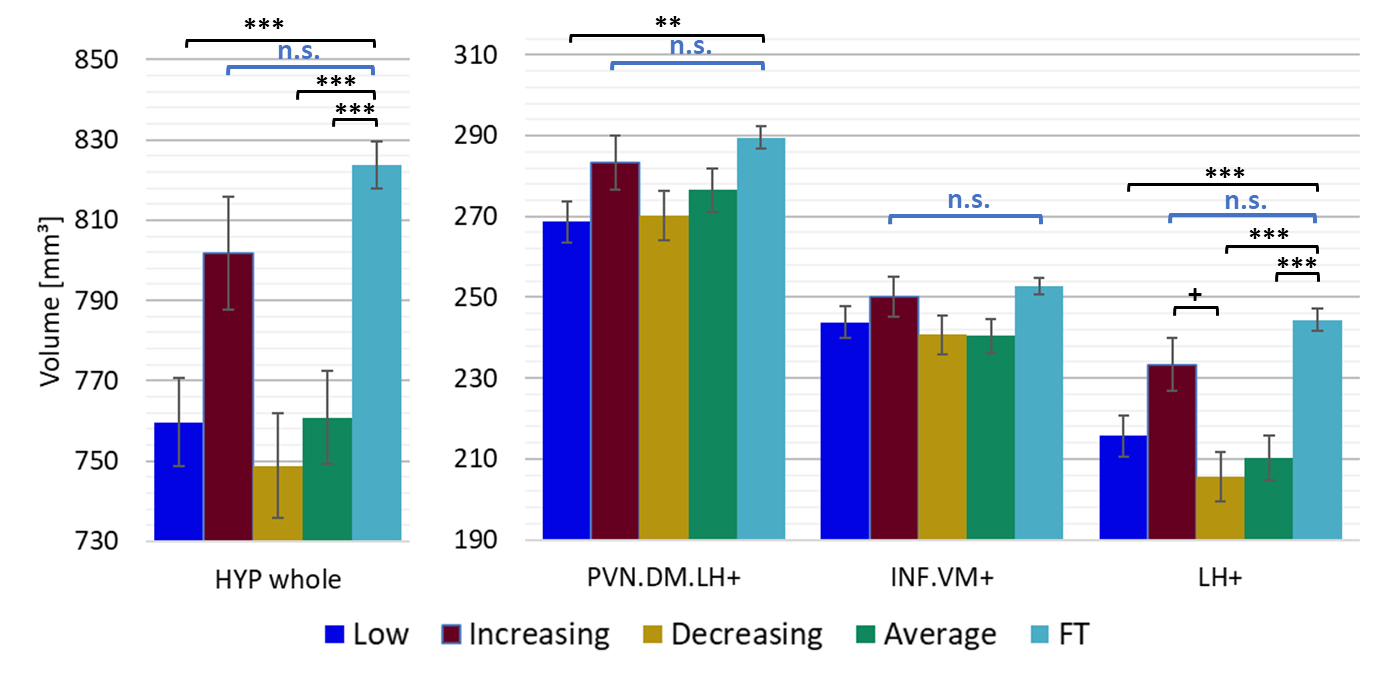

 **Figure S5.** Marginal means of hypothalamic volumes are given in mm³ for FT cohort and k-means clustered groups of VP/VLBW cohort and are shown as bar plots; error bars indicate SE. Group differences were assessed using a general linear model (fixed factor: five-part variable considering k-means clustered VP/VLBW and FT group differentiation; covariates of no interest: sex, scanners, TIV). Group difference significance is marked by asterisks (+: p < 0.05; *: p-FDR < 0.05; **: p-FDR < 0.01; *** p-FDR < 0.001).
Abbreviations: FDR, false discovery rate correction for multiple comparisons using the Benjamini–Hochberg method; FT, full-term; n.s., not significant; SE, standard error; VP/VLBW, very preterm and/or very low birth weight.

# Supplementary Tables

## Table S1: Intraventricular hemorrhage

|  | **VP/VLBW (n=101)** | |
| --- | --- | --- |
|  | **n** | **%** |
| **Intraventricular hemorrhage**  **None**  **Stage 1**  **Stage 2**  **Stage 3**  **Stage 4** | 85  5  7  3  1 | 84.2  5.0  6.9  3.0  1.0 |

**Table S1.** Number of VP/VLBW subjects with intraventricular hemorrhage and its grade assessed with ultrasound examinations in the neonatal period, graded 1-4.
Abbreviations: MRI, magnetic resonance imaging; VP/VLBW, very preterm and/or very low birth weight.

## Table S2: Description of the variables comprising the INTI

| **Scale value** | **Scale label** |
| --- | --- |
| 1. **Care level** | |
| 0 | Normal care (i.e., that required by term-born infant) |
| 1 | Special care |
| 2 | Intensive observation |
| 3 | Intensive treatment |
| 1. **Respiratory support** | |
| 0 | Air |
| 1 | Extra oxygen |
| 2 | CPAP |
| 3 | Ventilator |
| 1. **Feeding dependency** | |
| 0 | Oral |
| 1 | Gavage/ probe |
| 2 | Intravenous plus gavage and/or oral |
| 3 | Intravenous |
| 1. **Mobility** | |
| 0 | Normal |
| 1 | *No label specified* |
| 2 | Slightly diminished or elevated |
| 3 | Extremely diminished or elevated (hyper- or hypokinetic) |
| 1. **Muscle tone** | |
| 0 | Normal |
| 1 | *No label specified* |
| 2 | Slightly degraded or elevated |
| 3 | Hypertonic or hypotonic |
| 1. **Neurological excitability (of the Central Nervous System)** | |
| 0 | Normal |
| 1 | *No label specified* |
| 2 | Slightly delayed / weak or increased reactions possibly with trembling |
| 3 | Pronounced, decreased (apathy, coma) or severe hyperexcitability with abnormal signs |

**Table S2.** Description of the six variables that comprise the INTI.
Abbreviations: CPAP, continuous positive airway pressure; INTI, intensity of neonatal treatment index.

## Table S3: Hypothalamus parcellation methodologies and segmentation results in literature

**Table S3.** Comparison of manually or (semi-)automated segmented hypothalamic volumes (in cm³) in research literature, their parcellation methodology and segmentation approach for whole hypothalamus and hypothalamus subunits. For visualization of hypothalamic volume comparisons see Supplement Fig. S2. More examples of whole hypothalamus segmentation, parcellation methodologies and volumetric data in literature can be found in (36–38), from where some of the data in the table is also extracted from.
Abbreviations: approx., approximated; ADNI, Alzheimer’s Disease Neuroimaging Initiative; HCP, Human Connectome Project; MB excl., mammillary bodies excluded; SD, standard deviation; subsgm., subsegment; VAR coeff., variation coefficient; VP/VLBW, very preterm and/or very low birth weight.

## Table S4: Group comparison of hypothalamic volumes with respect to gray matter volume (instead of TIV) as covariate of no interest

|  | **VP/VLBW (n=101)** | | |  | **FT (n= 110)** | |  |  |  | |
| --- | --- | --- | --- | --- | --- | --- | --- | --- | --- | --- |
|  | **M** | **SE** | **95% CI** | | **M** | **SE** | **95% CI** | | **p-value** | |
| HYP whole (mm³) | 764.4 | 6.5 | 751.6 | 777.1 | 824.1 | 6.2 | 811.9 | 836.2 | <0.001*** |  |
| PVN.DM.LH^+^ (mm³) | 273.1 | 2.9 | 267.4 | 278.9 | 289.6 | 2.8 | 284.1 | 295.1 | <0.001*** | |
| INF.VM^+^ (mm³) | 242.0 | 2.3 | 237.4 | 246.5 | 253.3 | 2.2 | 249.0 | 257.7 | <0.001*** | |
| LH^+^ (mm³) | 216.4 | 2.9 | 210.6 | 222.2 | 244.1 | 2.8 | 238.5 | 249.6 | <0.001*** | |

**Table S4.** Marginal mean values of whole hypothalamus and its subunits are given in mm³. General linear models with prematurity status at birth as fixed factor and scanner, sex, and brain’s gray matter volume (instead of TIV) as covariates of no interest. Group difference significance is marked by asterisks (+: p < 0.05; *: p-FDR < 0.05; **: p-FDR < 0.01; *** p-FDR < 0.001).
Abbreviations: CI, confidence interval; FDR, false discovery rate correction for multiple comparisons using the Benjamini–Hochberg method; FT, full-term; M, mean; SE, standard error; VP/VLBW, very preterm and/or very low birth weight.

## Table S5: Group comparison of hypothalamic volumes with regards to sex differences

| A. Hypothalamic volumes (**VP/VLBW cohort**) | | | | | | | | | | |
| --- | --- | --- | --- | --- | --- | --- | --- | --- | --- | --- |
|  | **male (n=58)** | |  |  | **female (n= 43)** | |  |  |  |  |
|  | **M** | **SE** | **95% CI** | | **M** | **SE** | **95% CI** | | **p-value** |  |
| HYP whole (mm³) | 764.3 | 10.3 | 743.8 | 784.8 | 744.7 | 12.5 | 719.9 | 769.4 | 0.284 |  |
| PVN.DM.LH^+^ (mm³) | 275.0 | 4.0 | 266.9 | 283.0 | 265.0 | 4.9 | 255.3 | 274.6 | 0.163 |  |
| INF.VM^+^ (mm³) | 243.8 | 3.5 | 236.9 | 250.6 | 235.4 | 4.2 | 227.1 | 243.7 | 0.172 |  |
| LH^+^ (mm³) | 212.6 | 5.0 | 202.7 | 222.4 | 212.2 | 6.0 | 200.3 | 224.1 | 0.968 |  |
|  |  |  |  |  |  |  |  |  |  |  |
| B. Hypothalamic volumes (**FT cohort**) | | | | | | | | | | |
|  |  |  |  |  |  |  |  |  |  |  |
|  | **male (n=65)** | |  |  | **female (n= 45)** | |  |  |  |  |
|  | **M** | **SE** | **95% CI** | | **M** | **SE** | **95% CI** | | **p-value** |  |
| HYP whole (mm³) | 841.6 | 7.2 | 827.2 | 855.9 | 817.7 | 9.2 | 799.5 | 835.9 | 0.074 |  |
| PVN.DM.LH^+^ (mm³) | 296.1 | 4.0 | 288.2 | 304.1 | 285.7 | 5.1 | 275.6 | 295.8 | 0.159 |  |
| INF.VM^+^ (mm³) | 256.8 | 2.8 | 251.2 | 262.4 | 252.3 | 3.6 | 245.2 | 259.4 | 0.388 |  |
| LH^+^ (mm³) | 252.0 | 3.3 | 245.3 | 258.6 | 241.6 | 4.2 | 233.2 | 250.0 | 0.093 |  |

**Table S5.** Marginal mean values of whole hypothalamus and its subunits are given in mm³. General linear model with sex as fixed factor. Scanner and TIV served as covariates of no interest. Group difference significance is marked by asterisks (+: p < 0.05; *: p-FDR < 0.05; **: p-FDR < 0.01; *** p-FDR < 0.001).
Abbreviations: CI, confidence interval; FT, full-term; M, mean; SE, standard error; VP/VLBW, very preterm and/or very low birth weight.

## Table S6: Group comparison of hypothalamic volumes with regards to hypothalamic hemisphere

| A. Hypothalamic volumes (**VP/VLBW cohort**) | | | | | | | | | | |
| --- | --- | --- | --- | --- | --- | --- | --- | --- | --- | --- |
|  |  |  |  |  |  |  |  |  |  |  |
|  | **left hemisphere (n=101)** | | |  | **right hemisphere (n=101)** | | | |  | |
|  | **M** | **SE** | **95% CI** | | **M** | **SE** | **95% CI** | | **p-value** |  |
| HYP whole (mm³) | 386.4 | 3.6 | 379.4 | 393.4 | 369.5 | 3.6 | 362.5 | 376.5 | 0.001** |  |
| PVN.DM.LH^+^ (mm³) | 138.7 | 1.5 | 135.7 | 141.6 | 132.0 | 1.5 | 129.1 | 135.0 | 0.002** |  |
| INF.VM^+^ (mm³) | 123.0 | 1.2 | 120.6 | 125.4 | 117.2 | 1.2 | 114.8 | 119.6 | 0.001** |  |
| LH^+^ (mm³) | 107.7 | 1.7 | 104.3 | 111.1 | 104.7 | 1.7 | 101.3 | 108.1 | 0.210 |  |
|  |  |  |  |  |  |  |  |  |  |  |
| B. Hypothalamic volumes (**FT cohort**) | | | | | | | | | | |
|  |  |  |  |  |  |  |  |  |  |  |
|  | **left hemisphere (n=110)** | | |  | **right hemisphere (n=110)** | | | |  | |
|  | **M** | **SE** | **95% CI** | | **M** | **SE** | **95% CI** | | **p-value** |  |
| HYP whole (mm³) | 423.4 | 2.6 | 418.3 | 428.5 | 408.4 | 2.6 | 403.3 | 413.6 | <0.001*** |  |
| PVN.DM.LH^+^ (mm³) | 148.3 | 1.5 | 145.4 | 151.2 | 143.6 | 1.5 | 140.6 | 146.5 | 0.025* |  |
| INF.VM^+^ (mm³) | 130.7 | 1.1 | 128.6 | 132.8 | 124.2 | 1.1 | 122.1 | 126.3 | <0.001*** |  |
| LH^+^ (mm³) | 125.3 | 1.2 | 123.0 | 127.7 | 122.4 | 1.2 | 120.1 | 124.8 | 0.087 |  |

**Table S6.** Marginal mean values of whole hypothalamus and its subunits are given in mm³. General linear model with sidedness of hemisphere as fixed factor. Scanner, sex and TIV served as covariates of no interest. Group difference significance is marked by asterisks (+: p < 0.05; *: p-FDR < 0.05; **: p-FDR < 0.01; *** p-FDR < 0.001).
Abbreviations: CI, confidence interval; FT, full-term; M, mean; SE, standard error; VP/VLBW, very preterm and/or very low birth weight.

## Table S7: Relationship between hypothalamic volumes in VP/VLBW adults and variables of preterm birth

|  | **GA** |  |  | **INTI** |  |
| --- | --- | --- | --- | --- | --- |
|  | **r** | **p-Value** |  | **r** | **p-Value** |
| HYP whole | 0.265 | 0.009** |  | -0.296 | 0.004** |
| PVN.DM.LH**^+^** | 0.143 | 0.166 |  | -0.328 | 0.001** |
| INF.VM**^+^** | 0.247 | 0.016* |  | -0.141 | 0.174 |
| LH**^+^** | 0.236 | 0.021* |  | -0.251 | 0.014* |

**Table S7.** Correlation coefficients from partial correlation analyses in the VP/VLBW sample are given. TIV, scanner, and sex served as covariates. Group difference significance is marked by asterisks (+: p < 0.05; *: p-FDR < 0.05; **: p-FDR < 0.01; *** p-FDR < 0.001).
Abbreviations: FDR, false discovery rate correction for multiple comparisons using the Benjamini–Hochberg method; GA, gestational age; INTI, intensity of neonatal treatment; VP/VLBW, very preterm and/or very low birth weight.

## Table S8: Relationship between hypothalamic volumes in VP/VLBW adults and adult body weight

|  | **Adult body weight** | |
| --- | --- | --- |
|  | **r** | **p-Value** |
| HYP whole | 0.164 | 0.118 |
| PVN.DM.LH**^+^** | 0.214 | 0.041^+^ |
| INF.VM**^+^** | -0.035 | 0.740 |
| LH**^+^** | 0.098 | 0.352 |

**Table S8.** Correlation coefficients from partial correlation analyses in the VP/VLBW sample are given. TIV, scanner, and sex served as covariates. Group difference significance is marked by asterisks (+: p < 0.05; *: p-FDR < 0.05; **: p-FDR < 0.01; *** p-FDR < 0.001).
Abbreviations: FDR, false discovery rate correction for multiple comparisons using the Benjamini–Hochberg method; VP/VLBW, very preterm and/or very low birth weight.

## Table S9: Relationship between hypothalamic volumes in VP/VLBW adults and long-term delta slope of body weight trajectories

|  | **Long-term delta slope** | |
| --- | --- | --- |
|  | **r** | **p-Value** |
| HYP whole | 0.238 | 0.022^+^ |
| PVN.DM.LH**^+^** | 0.223 | 0.033^+^ |
| INF.VM**^+^** | 0.003 | 0.976 |
| LH**^+^** | 0.216 | 0.039^+^ |

**Table S9.** Correlation coefficients from partial correlation analyses in the VP/VLBW sample are given. Long-term delta slope of individual body weight trajectories is calculated via change of body weight z-score from birth until adulthood of regressed body weight trajectories. TIV, scanner, and sex served as covariates. Group difference significance is marked by asterisks (+: p < 0.05; *: p-FDR < 0.05; **: p-FDR < 0.01; *** p-FDR < 0.001).
Abbreviations: FDR, false discovery rate correction for multiple comparisons using the Benjamini–Hochberg method; VP/VLBW, very preterm and/or very low birth weight.

## Table S10: Group comparisons of hypothalamic volumes in the VP/VLBW cohort with regards to early development of body weight

|  | **"early catch-up" (n=19)** | | | | **"early catch-down" (n=33)** | | | |  |
| --- | --- | --- | --- | --- | --- | --- | --- | --- | --- |
|  | **M** | **SE** | **95% CI** | | **M** | **SE** | **95% CI** | | **p-value** |
| HYP whole (mm³) | 783.6 | 15.7 | 752.3 | 814.8 | 735.3 | 11.8 | 712.0 | 758.7 | 0.016^+^ |
| PVN.DM.LH^+^ (mm³) | 280.0 | 6.3 | 267.5 | 292.4 | 267.8 | 4.7 | 258.4 | 277.1 | 0.123 |
| INF.VM^+^ (mm³) | 249.8 | 5.3 | 239.2 | 260.4 | 236.2 | 4.0 | 228.2 | 244.1 | 0.045^+^ |
| LH^+^ (mm³) | 220.6 | 7.4 | 205.9 | 235.4 | 198.8 | 5.5 | 187.8 | 209.8 | 0.020^+^ |
|  |  |  |  |  |  |  |  |  |  |
|  | **"early catch-up" (n=19)** | | | | **"stable" (n=45)** | | | |  |
|  | **M** | **SE** | **95% CI** | | **M** | **SE** | **95% CI** | | **p-value** |
| HYP whole (mm³) | 783.6 | 15.7 | 752.3 | 814.8 | 760.1 | 10.1 | 739.9 | 780.2 | 0.218 |
| PVN.DM.LH^+^ (mm³) | 280.0 | 6.3 | 267.5 | 292.4 | 270.0 | 4.1 | 261.9 | 278.0 | 0.189 |
| INF.VM^+^ (mm³) | 249.8 | 5.3 | 239.2 | 260.4 | 240.6 | 3.4 | 233.8 | 247.5 | 0.159 |
| LH^+^ (mm³) | 220.6 | 7.4 | 205.9 | 235.4 | 217.2 | 4.8 | 207.7 | 226.7 | 0.699 |
|  |  |  |  |  |  |  |  |  |  |
|  | **"stable" (n=45)** | | | | **"early catch-down" (n=33)** | | | |  |
|  | **M** | **SE** | **95% CI** | | **M** | **SE** | **95% CI** | | **p-value** |
| HYP whole (mm³) | 760.1 | 10.1 | 739.9 | 780.2 | 735.3 | 11.8 | 712.0 | 758.7 | 0.117 |
| PVN.DM.LH^+^ (mm³) | 270.0 | 4.1 | 261.9 | 278.0 | 267.8 | 4.7 | 258.4 | 277.1 | 0.727 |
| INF.VM^+^ (mm³) | 240.6 | 3.4 | 233.8 | 247.5 | 236.2 | 4.0 | 228.2 | 244.1 | 0.404 |
| LH^+^ (mm³) | 217.2 | 4.8 | 207.7 | 226.7 | 198.8 | 5.5 | 187.8 | 209.8 | 0.014^+^ |

**Table S10.** Marginal mean values of whole hypothalamus and its subunits in the respective VP/VLBW cohorts are given in mm³. General linear model with early growth status in body weight (catch-up, catch-down, stable) as fixed factor. Scanner, sex and TIV served as covariates of no interest. Group difference significance is marked by asterisks (+: p < 0.05; *: p-FDR < 0.05; **: p-FDR < 0.01; *** p-FDR < 0.001).
Abbreviations: CI, confidence interval; M, mean; SE, standard error; VP/VLBW, very preterm and/or very low birth weight.

## Table S11: Relationship between hypothalamic volumes in VP/VLBW adults and short-term delta slope of body weight trajectories

|  | **Short-term delta slope** | |
| --- | --- | --- |
|  | **r** | **p-Value** |
| HYP whole | 0.329 | 0.001** |
| PVN.DM.LH**^+^** | 0.258 | 0.013* |
| INF.VM**^+^** | 0.232 | 0.026* |
| LH**^+^** | 0.282 | 0.006* |

**Table S11.** Correlation coefficients from partial correlation analyses in the VP/VLBW sample are given. Short-term delta slope of individual body weight trajectories is calculated via change of body weight z-score from birth until age of 20 months of regressed body weight trajectories. TIV, scanner, and sex served as covariates. Group difference significance is marked by asterisks (+: p < 0.05; *: p-FDR < 0.05; **: p-FDR < 0.01; *** p-FDR < 0.001).
Abbreviations: FDR, false discovery rate correction for multiple comparisons using the Benjamini–Hochberg method; VP/VLBW, very preterm and/or very low birth weight.

## Table S12: Relationship between hypothalamic volumes in VP/VLBW adults and birth weight z-scores

|  | **Birth weight z-score** | |
| --- | --- | --- |
|  | **r** | **p-Value** |
| HYP whole | -0.262 | 0.012* |
| PVN.DM.LH**^+^** | -0.082 | 0.438 |
| INF.VM**^+^** | -0.231 | 0.027* |
| LH^+^ | -0.273 | 0.009** |

**Table S12.** Correlation coefficients from partial correlation analyses in the VP/VLBW sample are given. TIV, scanner, and sex served as covariates. Group difference significance is marked by asterisks (+: p < 0.05; *: p-FDR < 0.05; **: p-FDR < 0.01; *** p-FDR < 0.001).
Abbreviations: FDR, false discovery rate correction for multiple comparisons using the Benjamini–Hochberg method; VP/VLBW, very preterm and/or very low birth weight.

## Table S13: Group comparison of hypothalamic volumes with regards to birth weight and status of preterm birth (VP/VLBW adults born SGA vs. VP/VLBW adults born AGA/LGA vs. adult FT)

|  | | **VP/VLBW SGA (n=30)** | | | | **VP/VLBW AGA/LGA (n= 67)** | | | |  |
| --- | --- | --- | --- | --- | --- | --- | --- | --- | --- | --- |
|  | | **M** | **SE** | **95% CI** | | **M** | **SE** | **95% CI** | | **p-value** |
| HYP whole (mm³) | | 790.8 | 10.9 | 769.3 | 812.2 | 753.9 | 7.5 | 739.1 | 768.8 | 0.016* |
| PVN.DM.LH^+^ (mm³) | | 279.3 | 5.1 | 269.3 | 289.3 | 271.6 | 3.5 | 264.7 | 278.6 | 0.627 |
| INF.VM^+^ (mm³) | | 249.6 | 3.9 | 242.0 | 257.3 | 240.5 | 2.7 | 235.2 | 245.8 | 0.150 |
| LH^+^ (mm³) | | 228.0 | 5.1 | 218.0 | 238.0 | 209.4 | 3.5 | 202.4 | 216.3 | 0.008** |
|  |  |  |  |  |  | |  |  |  |  |
|  | | **VP/VLBW SGA (n=30)** | | | | **FT (n=110)** | | | |  |
|  | | **M** | **SE** | **95% CI** | | **M** | **SE** | **95% CI** | | **p-value** |
| HYP whole (mm³) | | 790.8 | 10.9 | 769.3 | 812.2 | 823.8 | 5.9 | 812.3 | 835.4 | 0.027* |
| PVN.DM.LH^+^ (mm³) | | 279.3 | 5.1 | 269.3 | 289.3 | 289.4 | 2.7 | 284.0 | 294.8 | 0.262 |
| INF.VM^+^ (mm³) | | 249.6 | 3.9 | 242.0 | 257.3 | 252.8 | 2.1 | 248.7 | 257.0 | 0.999 |
| LH^+^ (mm³) | | 228.0 | 5.1 | 218.0 | 238.0 | 244.6 | 2.7 | 239.2 | 250.0 | 0.015* |
|  | |  |  |  |  |  |  |  |  |  |
|  | | **VP/VLBW AGA/LGA (n= 67)** | | | | **FT (n=110)** | | | |  |
|  | | **M** | **SE** | **95% CI** | | **M** | **SE** | **95% CI** | | **p-value** |
| HYP whole (mm³) | | 753.9 | 7.5 | 739.1 | 768.8 | 823.8 | 5.9 | 812.3 | 835.4 | <0.001*** |
| PVN.DM.LH^+^ (mm³) | | 271.6 | 3.5 | 264.7 | 278.6 | 289.4 | 2.7 | 284.0 | 294.8 | 0.001** |
| INF.VM^+^ (mm³) | | 240.5 | 2.7 | 235.2 | 245.8 | 252.8 | 2.1 | 248.7 | 257.0 | 0.002** |
| LH^+^ (mm³) | | 209.4 | 3.5 | 202.4 | 216.3 | 244.6 | 2.7 | 239.2 | 250.0 | <0.001*** |

**Table S13.** Marginal mean values of whole hypothalamus and its subunits are given in mm³. General linear model with SGA, AGA/LGA and FT group differentiation status at birth as fixed factor. Scanner, sex, and TIV served as covariates of no interest. Group difference significance is marked by asterisks (+: p < 0.05; *: p-FDR < 0.05; **: p-FDR < 0.01; *** p-FDR < 0.001).
Abbreviations: AGA/LGA, appropriate for gestational age/ large for gestational age; CI, confidence interval; FDR, false discovery rate correction for multiple comparisons using the Benjamini–Hochberg method; FT, full-term; M, mean; SE, standard error; SGA, small for gestational age; VP/VLBW, very preterm and/or very low birth weight.

# Supplementary References

1. Wolke D, Ratschinski G, Ohrt B, Riegel K. The cognitive outcome of very preterm infants may be poorer than often reported: an empirical investigation of how methodological issues make a big difference. Eur J Pediatr 1994; 153(12):906–15.

2. Wolke D, Meyer R. Cognitive status, language attainment, and prereading skills of 6-year-old very preterm children and their peers: the Bavarian Longitudinal Study. Dev Med Child Neurol 1999; 41(2):94–109.

3. Hedderich DM, Boeckh-Behrens T, Bäuml JG, Menegaux A, Daamen M, Zimmer C et al. Sequelae of Premature Birth in Young Adults : Incidental Findings on Routine Brain MRI. Clin Neuroradiol 2021; 31(2):325–33.

4. Schmitz-Koep B, Zimmermann J, Menegaux A, Nuttall R, Bäuml JG, Schneider SC et al. Decreased amygdala volume in adults after premature birth. Sci Rep 2021; 11(1):5403.

5. Dubowitz LM, Dubowitz V, Goldberg C. Clinical assessment of gestational age in the newborn infant. J Pediatr 1970; 77(1):1–10.

6. Casaer, P., Eggermont, E. Neonatal clinical neurological assessment. In: Harel S, Anastasiow N, editors. The at-risk infant: Psycho/- socio/medical aspects. Baltimore, MD: Brookes; 1985. pp. 197–220.

7. Bauer A. Ein Verfahren zur Messung des für das Bildungsverhalten relevanten Sozial Status (BRSS)—überarbeitete Fassung. Frankfurt: Deutsches Institut fuer Internationale Paedagogische Forschung; 1988.

8. Gutbrod T, Wolke D, Soehne B, Ohrt B, Riegel K. Effects of gestation and birth weight on the growth and development of very low birthweight small for gestational age infants: a matched group comparison. Arch Dis Child Fetal Neonatal Ed 2000; 82(3):F208-14.

9. Wang Y, Chen H-J. Use of Percentiles and Z-Scores in Anthropometry. In: Preedy VR, editor. Handbook of Anthropometry: Physical Measures of Human Form in Health and Disease. 2012nd ed. Dordrecht: New York: Springer; 2012.

10. Wit J-M, Boersma B. Catch-up growth: definition, mechanisms, and models. J Pediatr Endocrinol Metab 2002; 15 Suppl 5:1229–41.

11. Eves R, Mendonça M, Bartmann P, Wolke D. Small for gestational age-cognitive performance from infancy to adulthood: an observational study. BJOG 2020; 127(13):1598–606.

12. Voigt M, Fusch C, Olbertz D, Hartmann K, Rochow N, Renken C et al. Analyse des Neugeborenenkollektivs der Bundesrepublik Deutschland. Geburtsh Frauenheilk 2006; 66(10):956–70.

13. Kromeyer-Hauschild K, Wabitsch M, Kunze D, Geller F, Geiß HC, Hesse V et al. Perzentile für den Body-mass-Index für das Kindes- und Jugendalter unter Heranziehung verschiedener deutscher Stichproben. Monatsschr Kinderheilkd 2001; 149(8):807–18. Available from: URL: https://link.springer.com/article/10.1007/s001120170107.

14. Statistisches Bundesamt. Fragen zur Gesundheit - Körpermaße der Bevölkerung - Mikrozensus 2017; 2017 [cited 2021 Jun 22]. Available from: URL: https://www.destatis.de/DE/Themen/Gesellschaft-Umwelt/Gesundheit/Gesundheitszustand-Relevantes-Verhalten/Publikationen/Downloads-Gesundheitszustand/koerpermasse-5239003179004.html.

15. Physical status: the use and interpretation of anthropometry. Report of a WHO Expert Committee. World Health Organ Tech Rep Ser 1995; 854:1–452.

16. Louise T, Nauf Bendar AS, Durighel G, Frost G, Bell J. The effect of preterm birth on adiposity and metabolic pathways and the implications for later life. Clinical Lipidology 2012; 7(3):275–88.

17. Euser AM, Wit CC de, Finken MJJ, Rijken M, Wit J-M. Growth of preterm born children. Horm Res 2008; 70(6):319–28.

18. Olbertz DM, Mumm R, Wittwer-Backofen U, Fricke-Otto S, Pyper A, Otte J et al. Identification of growth patterns of preterm and small-for-gestational age children from birth to 4 years - do they catch up? J Perinat Med 2019; 47(4):448–54.

19. Toftlund LH, Halken S, Agertoft L, Zachariassen G. Catch-Up Growth, Rapid Weight Growth, and Continuous Growth from Birth to 6 Years of Age in Very-Preterm-Born Children. Neonatology 2018; 114(4):285–93.

20. Fewtrell MS, Doherty C, Cole TJ, Stafford M, Hales CN, Lucas A. Effects of size at birth, gestational age and early growth in preterm infants on glucose and insulin concentrations at 9-12 years. Diabetologia 2000; 43(6):714–7.

21. Lapillonne A, Griffin IJ. Feeding preterm infants today for later metabolic and cardiovascular outcomes. J Pediatr 2013; 162(3 Suppl):S7-16.

22. Jain AK. Data clustering: 50 years beyond K-means. Pattern Recognition Letters 2010; 31(8):651–66. Available from: URL: https://www.sciencedirect.com/science/article/pii/S0167865509002323.

23. Likas A, Vlassis N, J. Verbeek J. The global k-means clustering algorithm. Pattern Recognition 2003; 36(2):451–61.

24. Lloyd S. Least squares quantization in PCM. IEEE Transactions on Information Theory 1982; 28(2):129–37.

25. Aster M von, Neubauer A, Horn R. Wechsler Intelligenztest für Erwachsene (WIE) [Wechsler Adult Intelligence Scale (WAIS III)] - Deutschsprachige Bearbeitung und Adaptation des WAIS-III von David Wechsler (3rd ed.). Frankfurt, Germany: Pearson; 2006.

26. Hedderich DM, Avram M, Menegaux A, Nuttall R, Zimmermann J, Schneider SC et al. Hippocampal subfield volumes are nonspecifically reduced in premature-born adults. Hum Brain Mapp 2020; 41(18):5215–27.

27. Billot B, Bocchetta M, Todd E, Dalca AV, Rohrer JD, Iglesias JE. Automated segmentation of the hypothalamus and associated subunits in brain MRI. Neuroimage 2020; 223:117287.

28. Ronneberger O, Fischer P, Brox T. U-Net: Convolutional Networks for Biomedical Image Segmentation. In: Medical image computing and computer-assisted intervention - MICCAI 2015: 18th International Conference, Munich, Germany, October 5-9, 2015, proceedings / Nassir Navab, Joachim Hornegger, William M. Wells, Alejandro F. Frangi (eds.). Cham: Springer; 2015. p. 234–41 (LNCS sublibrary: SL6 - Image processing, computer vision, pattern recognition, and graphics; 9349-9351).

29. Rodrigues L, Rezende T, Zanesco A, Hernandez AL, Franca M, Rittner L. Hypothalamus fully automatic segmentation from MR images using a U-Net based architecture. In: 15th International Symposium on Medical Information Processing and Analysis: 6-8 November 2019, Medellín, Colombia. Bellingham, Washington: SPIE; 2020. p. 40 (Proceedings of SPIE. 5200-; volume 11330). Available from: URL: https://www.spiedigitallibrary.org/conference-proceedings-of-spie/11330/2542585/Hypothalamus-fully-automatic-segmentation-from-MR-images-using-a-U/10.1117/12.2542585.full.

30. Hayes AF. Introduction to mediation, moderation, and conditional process analysis: A regression-based approach. Second edition. New York, New York, London [England]: New York, NY: The Guilford Press; 2018. (Methodology in the social sciences).

31. Altman DG, Bland JM. How to obtain the P value from a confidence interval. BMJ 2011; 343:d2304.

32. Benjamini Y, Hochberg Y. Controlling the False Discovery Rate: A Practical and Powerful Approach to Multiple Testing. Journal of the Royal Statistical Society. Series B (Methodological) 1995; 57(1):289–300. Available from: URL: http://www.jstor.org/stable/2346101.

33. Bocchetta M, Gordon E, Manning E, Barnes J, Cash DM, Espak M et al. Detailed volumetric analysis of the hypothalamus in behavioral variant frontotemporal dementia. J Neurol 2015; 262(12):2635–42.

34. Neudorfer C, Germann J, Elias GJB, Gramer R, Boutet A, Lozano AM. A high-resolution in vivo magnetic resonance imaging atlas of the human hypothalamic region. Sci Data 2020; 7(1):305.

35. Makris N, Swaab DF, van der Kouwe A, Abbs B, Boriel D, Handa RJ et al. Volumetric parcellation methodology of the human hypothalamus in neuroimaging: normative data and sex differences. Neuroimage 2013; 69:1–10.

36. Spindler M, Özyurt J, Thiel CM. Automated diffusion-based parcellation of the hypothalamus reveals subunit-specific associations with obesity. Sci Rep 2020; 10(1):22238.

37. Schindler S, Schönknecht P, Schmidt L, Anwander A, Strauß M, Trampel R et al. Development and evaluation of an algorithm for the computer-assisted segmentation of the human hypothalamus on 7-Tesla magnetic resonance images. PLoS One 2013; 8(7):e66394.

38. Gabery S, Georgiou-Karistianis N, Lundh SH, Cheong RY, Churchyard A, Chua P et al. Volumetric analysis of the hypothalamus in Huntington Disease using 3T MRI: the IMAGE-HD Study. PLoS One 2015; 10(2):e0117593.
